# Validation of intrinsic capacity and healthy sleep pattern in middle-aged and older adults: a longitudinal Chinese study assessing healthy ageing

**DOI:** 10.1016/j.jnha.2024.100365

**Published:** 2024-09-21

**Authors:** Xing-Ling Chen, Jin Li, Shu-Ning Sun, Xiao-Jiao Zhang, Jia-Hui Chen, Ling-Jun Wang, Zhong-Qi Yang, Shi-Hao Ni, Lu Lu

**Affiliations:** a State Key Laboratory of Traditional Chinese Medicine Syndrome, The First Affiliated Hospital, Guangzhou University of Chinese Medicine, Guangzhou 510407, China; b Lingnan Medical Research Center, Guangzhou University of Chinese Medicine, Guangzhou 510407, China; c University Key Laboratory of Traditional Chinese Medicine Prevention and Treatment of Chronic Heart Failure, Guangdong Province 510407, China; d Guangzhou Key Laboratory for Chinese Medicine Prevention and Treatment of Chronic Heart Failure, Guangzhou University of Chinese Medicine, Guangzhou 510407, China

**Keywords:** IC, Psychological, Cognitive, Locomotion, Nap, Functional performance

## Abstract

**Objectives:**

Intrinsic capacity (IC), a multidimensional construct encompassing mental and physical capacities, has been established in the aging framework by the World Health Organization. However, the detailed relationship between IC and Chinese sleep patterns (nighttime sleep and post-lunch naps) remains inadequately elucidated.

**Methods:**

Participants in this study were individuals aged ≥45 years residing in China, included in the China Health and Retirement Longitudinal Study (CHARLS). We analyzed 4 years of CHARLS data from the first wave (May 2011–March 2012) to the second wave (July 2015–January 2016). Data from these waves were utilized for longitudinal analysis. Self-reported data included nighttime sleep and nap duration, along with other baseline characteristics. The IC evaluation involved physical examinations and blood tests. Initially, linear regression was used to assess the relationship between total sleep duration, nighttime sleep duration, nap duration, and IC change between the two waves that were determined by marginal effects (ME) and their corresponding 95% confidence intervals (CIs). Regression splines were employed to explore potential nonlinear associations. Subgroup and sensitivity analyses were conducted to investigate the heterogeneity of IC change under specific conditions and the robustness of our results. Mediation analysis was performed to identify potential factors mediating the relationship between sleep patterns and IC change.

**Results:**

Both excessive (>10 h) (total, ME: −1.12; 95% CI: −1.61, −0.64; nighttime, ME: −1.44; 95% CI: −2.29, −0.59) and insufficient (<6 h) sleep duration (total, ME: −0.43; 95% CI: −0.68, −0.18; nighttime, ME: −0.50; 95% CI: −0.73, −0.27) negatively impacted IC change. Moderate naps (≤60 min) mitigated the decline in IC change (ME: 0.28; 95% CI: 0.07, 0.49). IC values decreased at the slowest rate when nap time constituted one-seventh of total sleep time. The onset of dyslipidemia partially mediated the association between naps (≤60 min) and IC change (*P* =  0.02).

**Conclusions:**

These findings suggest that maintaining a healthy sleep pattern of 6−8 h of nighttime or total sleep, along with a post-lunch nap of ≤60 min, helps preserve optimal IC or delay its decline. This is particularly beneficial for cognitive, psychological, and locomotion performance among middle-aged and older adults.

## 1 Introduction

Although populations worldwide are rapidly aging, there is little evidence to suggest that longer lifespans are accompanied by extended periods of good health [1]. Maintaining physical and mental well-being in old age is crucial for enhancing quality of life and enabling older adults to participate in desired activities without limitations [2]. However, if life extension is accompanied by a significant decline in both physical and cognitive abilities, it will negatively impact both individuals and society.

To address the challenges of population aging, the World Health Organization (WHO) proposes a function-based that evaluates healthy aging through an individual’s intrinsic capacity (IC), which reflects their physical and mental capacities, rather than their disease status [2]. IC provides a holistic and comprehensive framework for assessing and managing the healthcare needs of older adults and has been applied in several studies involving large and representative samples of older populations in the UK, China, and Brazil [[3], [4], [5], [6], [7], [8], [9]]. These studies have identified five core domains of IC: locomotion, sensory, vitality, cognitive, and psychological, which may reflect the biological drivers of the aging process. Moreover, three longitudinal studies have confirmed that IC is a strong predictor of functional quality of life and negative health outcomes in the future [[7], [8], [9]].

Sleep subject is widely discussed among the older population [10]. A growing body of epidemiological studies has demonstrated associations between various sleep habits, such as nighttime sleep, and daytime napping, and a range of detrimental health conditions, including memory impairments [11], mental health issues [12], and obesity [13]. Notably, in most previous studies among older adults, few large population-based studies specifically examined the independent association of total sleep duration, nocturnal sleep, and napping with IC changes. Additionally, a comprehensive and long-term assessment of the optimal ratio of sleep duration between day and night has been less studied.

Considering that China is one of the countries with a prevalent napping habit worldwide [14], with an estimated 58% of Chinese people often taking naps after lunch [15], it is important to examine the role of daytime napping in this context. Previous research has suggested that daytime naps can compensate for the effects of overall sleep length on the risk of cancer [16] or cardiovascular disease [14]. Sleep is a fundamental aspect of health that influences multiple domains of IC. Poor sleep patterns, including insufficient (<6 h) or excessive (>8 h) sleep, are associated with negative health outcomes such as cognitive decline [17], psychological distress [18], and reduced physical function [19]. Despite this, the relationship between specific sleep patterns and IC has not been fully elucidated. The individual relationships between nap time, nighttime sleep, total sleep duration, and IC remain unclear, and the optimal sleep pattern for the middle-aged and elderly also warrants further investigation.

This study hypothesizes that maintaining a balanced sleep pattern, particularly ensuring 6−8 h of nighttime sleep and moderate daytime napping, is crucial for preserving IC and preventing its decline. Our objective is to explore how different sleep patterns affect the five domains of IC and to identify which patterns are most beneficial for healthy aging. In addition, given the established associations between sleep patterns and various health outcomes [20,21], we hypothesized that the effects of sleep on IC might be mediated by specific health factors, leading us to conduct a mediation analysis.

## 2 Methods

### 2.1 Data and study participants

We used publicly available de-identified data from the China Health and Retirement Longitudinal Study (CHARLS) to measure the IC construct and to examine daytime, nighttime, and overall sleep duration within it. The CHARLS is a nationally representative longitudinal survey of Chinese residents aged 45 and older. The baseline national wave of CHARLS was fielded in 2011 and included about 150 counties/districts and 450 villages/resident committees. All respondents were invited to participate in the Life History Survey, which included a series of questions about background, health status and behaviors, health care utilization and expenditure, income and consumption, family and social networks, psychological well-being, and comprised measurements about physical examinations and biomarkers.

Our study included individuals aged 45–100 at baseline who completed at least one sleep questionnaire and had follow-up data, excluding those with over 40% missing IC data. Missing values were imputed using multiple imputation methods. Changes in IC were calculated for 2011 and 2015 due to available biomarker data in these years. A flowchart of participant selection is provided in Supplementary Material (Figure S1).

### 2.2 Exposure assessment

Sleep information was collected using standardized questions on nighttime sleep and post-meal napping. (a) During the past month, how many hours of actual sleep did you get at night (average hours for one night)? (This may be shorter than the number of hours you spend in bed); (b) During the past month, how long did you take a nap after lunch? The total daily sleep was calculated by summing nighttime sleep and napping duration. To avoid bias, we used the average of sleep data from 2011 and 2013. Nighttime sleep and napping were analyzed as critical components, and the ratio of daytime naps to nighttime sleep was calculated. Subgroup comparisons based on sleep patterns and IC changes were made. Additionally, we compared populations that adhere to this sleep mode with those who do not, to uncover potential differences in their functional abilities and IC changes.

### 2.3 Intrinsic capacity measurement

IC was measured using five subdomains: locomotion, sensory, vitality, psychological, and cognitive capacities, following established methods using structural equation modeling (SEM). Each subdomain is evaluated using various indicators, such as (1) locomotion: balance, walking speed time, and chair-stand test; (2) sensory: vision and hearing impairments; (3) vitality: forced expiratory volume, grip strength, and hemoglobin; (4) psychological capacity: ten-item Center for Epidemiological Studies-Depression (CES-D) scale (To avoid misleading correction, we did not include sleep length and sleep quality in the assessment); (5) cognitive capacity: immediate and delayed recall, drawing and math. Please refer to Supplementary Materials for specific measurement methods. The bi-factor SEM model fit the data well (RMSEA = 0.094, CFI = 0.898), and IC was analyzed as a standardized continuous variable.

### 2.4 Covariates

Covariates such as demographic variables, socioeconomic status, lifestyle behaviors, and health conditions were included to adjust for confounding factors. Demographic variables were collected, which included age, gender, residence, educational level, and married status. Socioeconomic status specifically refers to household annual income. Lifestyle behaviors included physical activity level (MET-PA), smoking status, drinking status, memory, mental health, taking tranquilizers or sleeping pills, and sleep quality. Health conditions contained physical disabilities 14 medically diagnosed conditions and body mass index BMI. Details on covariates are provided in Supplementary Material.

### 2.5 Descriptive statistics

Based on the recent consensus reached by the American Academy of Sleep Medicine (AASM) and the Sleep Research Society (SRS), it is recommended that adults have the best sleep time of 7 h or more per night [22]. We take 7 h as the median and 6−8 h of sleep time as a control reference. We categorized the estimated nighttime and total daily sleep duration into four groups (<6 h, 6–8 h which was used as the reference, 8–10 h, and >10 h), and categorized the estimated napping time into three groups (0 min, which was used as the reference, ≤60 min, >60 min). Continuous variables were expressed as mean (standard deviation), while categorical variables were presented as numbers (percentages). One-way analysis of variance (ANOVA) was utilized to compare the mean values of continuous variables, while the chi-square test was employed to analyze the percentage distribution of categorical variables. We used "rcs", "Publish", and "mediation" package in R version 3.6.1. Statistical significance was determined based on a two-sided *P* < 0.05.

### 2.6 Linear and restricted cubic spline regression

Multivariable linear regression models were used to evaluate the association of daytime, nighttime, and overall sleep patterns with IC. The observed correlation is represented by the marginal effects, which refer to the change in the dependent variable (IC) resulting from a one-unit change in an independent variable (such as sleep duration or nap time) while holding other variables constant. Marginal effects (ME) and their corresponding 95% confidence intervals (CIs) were determined for IC changes within its five subdomains. Restricted cubic splines were used to explore the shape of the association between estimated daily sleep patterns and the outcomes (created by SAS LGTPHCURV9 Macro).

### 2.7 Subgroup and sensitivity analyses

We conducted subgroup analysis and sensitivity analysis to validate our findings. To facilitate a clearer comparison, we will categorize the study participants into six distinct groups based on their sleep duration: individuals with total sleep time >10 h and <6 h, those with night sleep time >10 h and <6 h, participants engaging in napping ≤60 min. and >60 min. This approach will allow for a more nuanced analysis of the effects of varying sleep patterns on the observed outcomes.

For subgroup analysis, it is divided into the following five groups: (a) <60 years old / ≥60 years old (b) BMI (normal/overweight / obesity) (c) residence (rural/urban areas) (d) gender (male/female), and (e)the stratification of the median of IC in 2011 (above median/under median). In the sensitivity analysis, several steps were taken: (a)we excluded participants who reported using sedatives or sleeping pills; (b)physical disabilities were eliminated; (c) we also removed data points with extreme values of IC (add or subtract five times the standard deviation); (d) data points with a total sleep length value of zero were ruled out.

### 2.8 Mediation effect analysis

Our study investigated the mediating effects of changes in BMI and the occurrence of chronic diseases (refers to the onset of chronic disease that was diagnosed in 2013 or 2015 but not in 2011) on the associations between daytime, nighttime, all-day sleep patterns, and IC change. The grouping method is consistent with subgroup analysis and sensitivity analysis. The mediation analysis encompassed several procedures: (a) computed the linear relationship between BMI changes, chronic diseases, and IC. (b) a linear model was utilized to analyze the results, encompassing all variables about sleep pattern. (c) employed the mediation ratio determination methods outlined by Preacher, Kristopher J, and Hayes, Andrew F [23]. We established a 95% confidence interval using the bootstrap method and repeated the procedure 5000 times.

## 3 Result

### 3.1 Population characteristics

As shown in Supplementary Tables 1, our study comprised a total of 12826 participants. Individuals who slept >10 h per day were found to be older, male, had higher household income and reported good sleep quality compared to those with an estimated nighttime sleep duration of 6−8 h per day. Conversely, individuals who slept <6 h per day were more likely to be female, had lower socioeconomic status, less alcohol consumption, poorer sleep quality, reported symptoms of depression, and had higher ratios of kidney diseases, digestive diseases, and arthritis/rheumatism. There are also differences between groups with varying sleep durations, with participants exhibiting differences in education levels, physical activity, memory, and likelihood of residing in rural areas. It is worth noting that a majority of men in the study took naps lasting at least 60 min, and these individuals tended to be smokers and drinkers, with better sleep quality, while most women did not take naps, and their sleep quality was relatively poor.

### 3.2 Association sleep pattern with intrinsic capacity

**Fig. 1:**
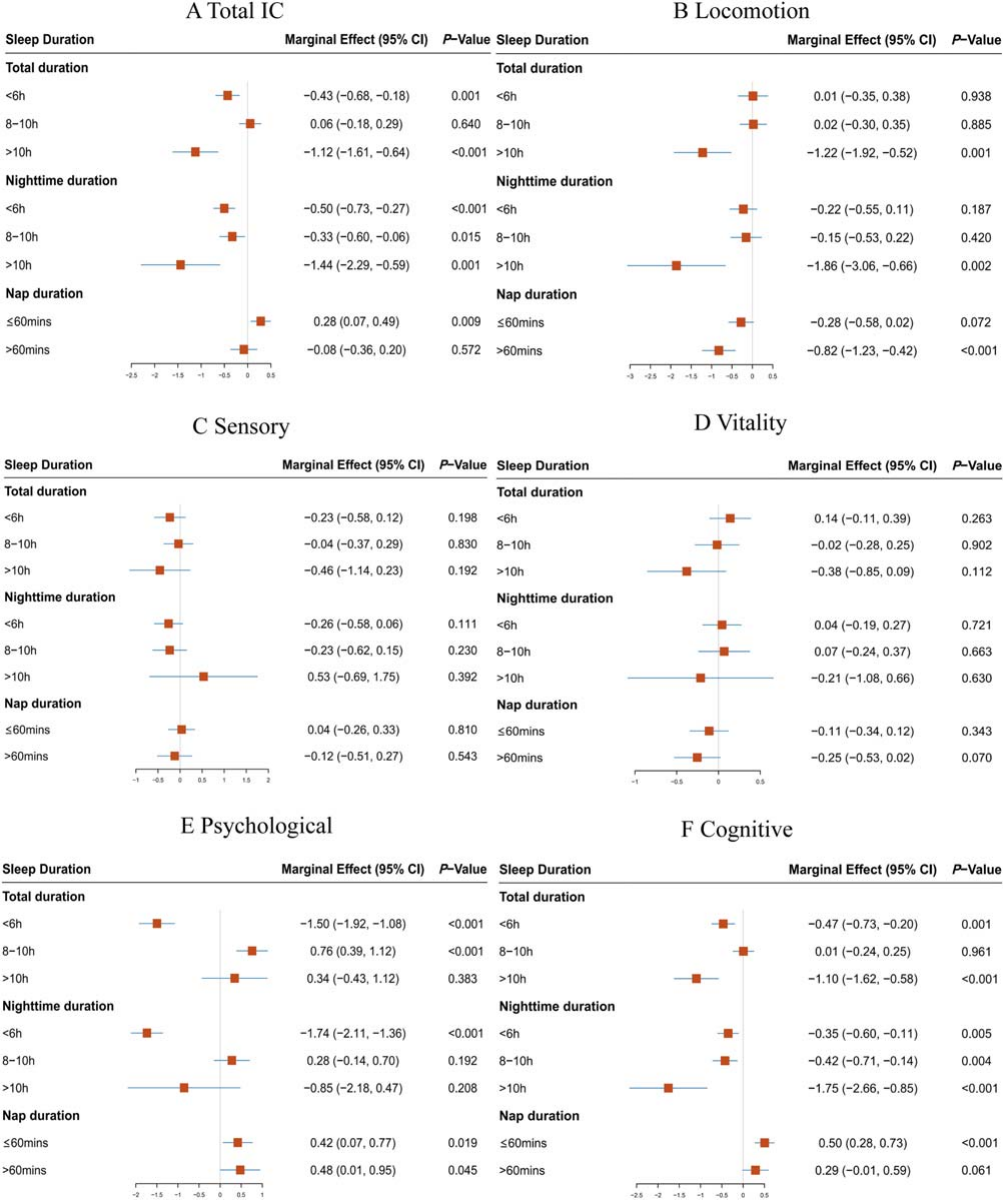
Linear regression analysis of multi-variable adjusted association between IC change with its five subdomains and sleep duration. 95%CI: 95% confidence intervals; IC: intrinsic capacity. h: hour; mins: minutes

After adjusting for 29 covariates encompassing demographic characteristics, lifestyle, socioeconomic status, sleep quality and medications, mental health, and health conditions, we examined the associations between sleep patterns (including sleep duration and the ratio of nap to night sleep) and changes in IC and its five subdomains, as depicted in Fig. 1.

Our findings suggest that individuals who maintain a sleep duration of 6−8 h of nighttime or total sleep, and a daytime nap duration of ≤60 min, experience the most beneficial effects on changes in IC. Specifically, compared to those with 6−8 h of sleep both at night and throughout the day, individuals with sleep durations exceeding 10 h showed a more rapid decline in IC (total sleep: ME, −1.12; 95% CI, −1.61 to −0.64; nighttime sleep: ME, −1.44; 95% CI, −2.29 to −0.59), particularly in cognitive capacity (total sleep: ME, −1.10; 95% CI, −1.62 to −0.58; nighttime sleep: ME, −1.75; 95% CI, −2.66 to −0.85) and locomotion (total sleep: ME, −1.22; 95% CI, −1.92 to −0.52; nighttime sleep: ME, −1.86; 95% CI, −3.06 to −0.66). Similarly, sleep durations of less than 6 h were associated with an accelerated decline in IC (total sleep: ME, −0.43; 95% CI, −0.68 to −0.18; nighttime sleep: ME, −0.50; 95% CI, −0.73 to −0.27), predominantly affecting psychological (total sleep: ME, −1.50; 95% CI, −1.92 to −1.08; nighttime sleep: ME, −1.74; 95% CI, −2.11 to −1.36) and cognitive capacity (total sleep: ME, −0.47; 95% CI, −0.73 to −0.20; nighttime sleep: ME, −0.35; 95% CI, −0.60 to −0.11).

Comparing middle-aged and older individuals who do not nap, a nap duration of ≤60 min (ME, 0.28; 95% CI, 0.07 to 0.49) was associated with optimal levels of IC change, particularly in psychological (ME, 0.42; 95% CI, 0.07 to 0.77) and cognitive (ME, 0.50; 95% CI, 0.28 to 0.73) capacities. However, napping for more than 60 min was detrimental to locomotion (ME, −0.82; 95% CI, −1.23 to −0.42). Additionally, all evaluation indicators for locomotion were negatively correlated with taking naps longer than 60 min and with total sleep durations exceeding 10 h. Detailed data for other evaluation indicators can be found in Figures S3-6.

### 3.3 Examination of the dose-response effect

**Fig. 2:**
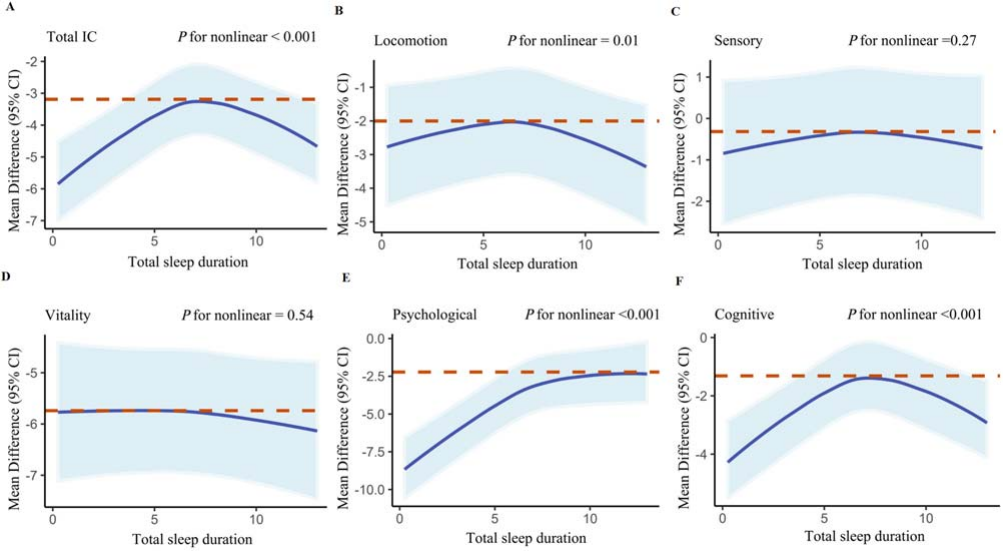
RCS models for the relationship between IC change with its five subdomains and the total sleep duration; RCS: restricted cubic spline; 95%CI: 95% confidence intervals; IC: intrinsic capacity.

**Fig. 3:**
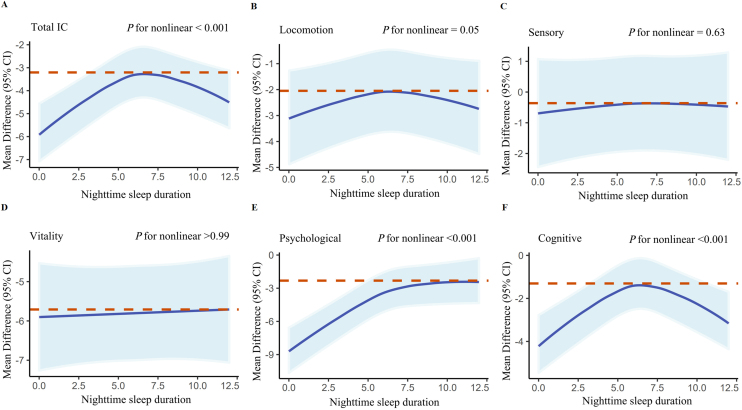
RCS models for the relationship between IC change with its five subdomains and the nighttime sleep duration; RCS: restricted cubic spline; 95%CI: 95% confidence intervals; IC: intrinsic capacity.

**Fig. 4:**
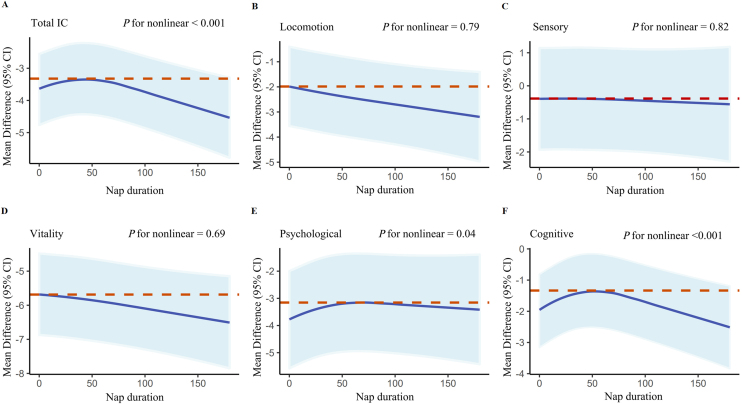
RCS models for the relationship between IC change with its five subdomains and the nap duration; RCS: restricted cubic spline; 95%CI: 95% confidence intervals; IC: intrinsic capacity.

To explore the trends in sleep duration and IC changes, as well as the optimal ratio of nap to night sleep, we performed a restricted cubic spline analysis. This method was employed to examine the relationship between IC changes and its five subdomains in relation to sleep duration (Fig. 2, Fig. 3, Fig. 4). The performance indicators of the five subdomains concerning IC changes are shown in Figures S7−10.

Our analysis revealed an inverted U-shaped relationship (non-linear *P*-value < 0.001) between IC change and sleep duration during naps, nighttime, and overall, indicating a positive correlation within specific ranges of sleep duration. However, no significant additional benefits were observed outside these ranges. Additionally, we found that IC values decreased at the slowest rate when nap time constituted one-seventh of total sleep time Figure S11.

**Fig. 5:**
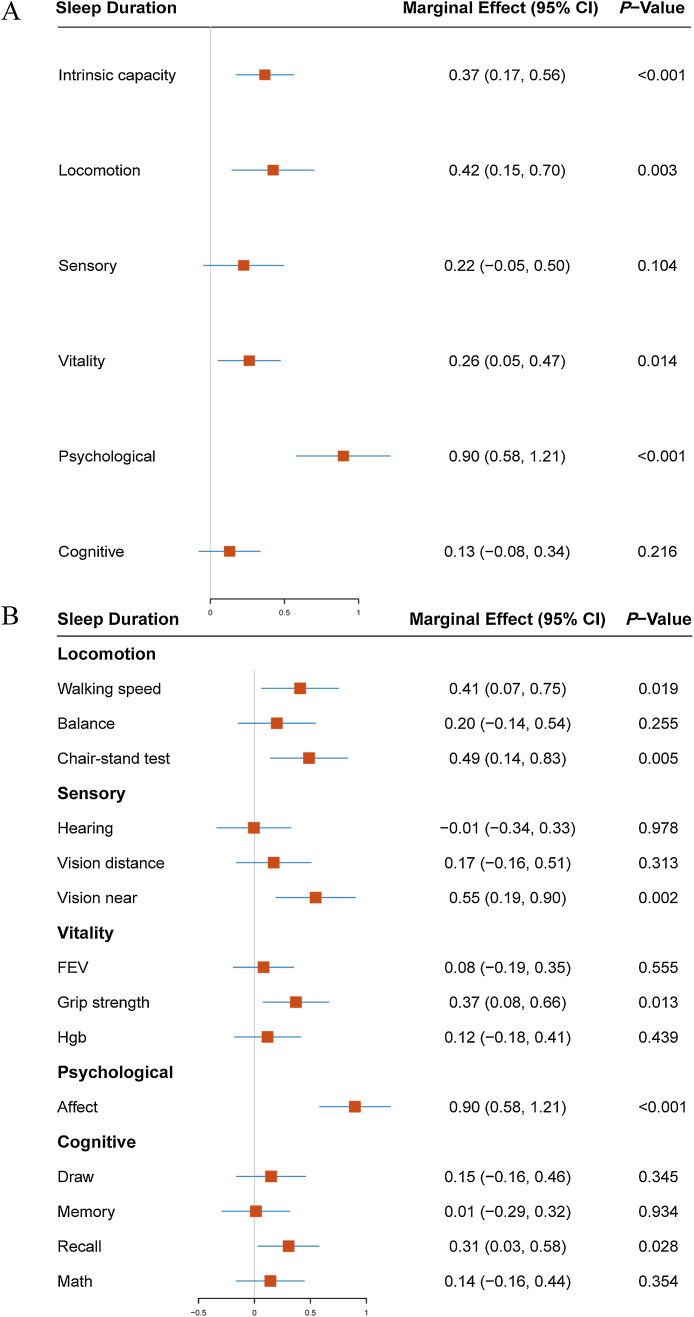
Linear regression analysis of multi-variable adjusted association between specific sleep patterns and IC changes with its five subdomains. 95%CI: 95% confidence intervals; IC: intrinsic capacity; FEV: forced expiratory volume; Hgb: hemoglobin.

We conducted a comparison between individuals who maintained a healthy sleep pattern (6−8 h of nighttime sleep and nap duration ≤60 min) and those who did not. Our findings revealed that among middle-aged and elderly populations, adhering to this healthy sleep routine resulted in a more favorable level of IC change (ME, 0.37; 95% CI, 0.17 to 0.56, *P* = 0.001), potentially increasing the likelihood of improving functional abilities and preventing aging (Fig. 5).

### 3.4 Subgroup analyses and sensitivity analyses

Various subgroup and sensitivity analyses were conducted to examine the robustness of our findings, as presented in Figure S12−17. The relationship between changes in psychological capacity and sleep was more pronounced in females than in males. Specifically, females who slept less than 6 h at night or overall experienced greater impairment in psychological capacity compared to those who slept 6−8 h, with a significant interaction effect (*P*-value for interaction = 0.003). In our subgroup analysis of napping (Figure S18), we observed some trends, such as a potential impairment in vitality among those with excessive nighttime sleep who also napped. However, these trends were not statistically significant across any of the subgroups (*P* > 0.05). In the sensitivity analysis, there remained no alteration in our results regarding the correlation between IC change and specific models. This proves the reliability of our results (Table S2−7).

### 3.5 Mediation analysis for 14 chronic diseases and BMI changes

To further investigate the underlying relevance of the association between sleep duration and IC, we employed a mediation analysis to examine the contributions of 14 chronic diseases and changes in BMI (Tables S8−13). The findings revealed that for nap durations of ≤60 min, dyslipidemia accounted for −3.37% of the total IC score change (*P* =  0.02), primarily due to its impact on psychological capacity, which accounted for −4.95% (*P* <  0.01). Thus, the potential benefits of proper napping on IC change might be achieved through the regulation of blood lipid stability. Currently, our results do not provide evidence to suggest that changes in BMI mediate the relationship between IC and sleep duration across the six groups.

## 4 Discussion

This cohort study first focused on the association between sleep patterns and IC change in Chinese older people. It was observed that maintaining the sleep pattern of 6−8 h of night or total sleep, along with daytime nap duration ≤60 min helps preserve optimal IC change or delay IC decline in middle-aged and older adults. The observed associations were consistent and remained robust after adjustment for 29 covariates. We also found dyslipidemia onset partially mediates the association between naps (less than 60 min) and IC change.

Biological aging, driven by underlying biological mechanisms, contributes to the decline in IC as individuals grow older. This decline particularly impacts the sensory, vitality, and psychological domains of IC, underscoring the multifaceted nature of aging. Biological aging is a comprehensive process that accounts for the variability in functional decline among individuals, extending beyond just physical and cognitive functions [1]. It also encompasses sensory, vitality, and psychological dimensions, which are essential components of IC. [24]. These five domains collectively represent critical considerations for older individuals in their daily lives [2]. By effectively identifying diverse levels of functional capacity, IC demonstrates its ability to capture the heterogeneity present within the older population, independent of clinical phenotypes [5]. IC as a composite measure, provides a more multifaceted framework for assessing healthy aging by integrating these various domains of functional capacity [1]. This broader approach allows for a more detailed understanding of how aging impacts overall health beyond the traditional focus on physical and cognitive decline [2].

Analyzing the aging situation of China's older population has important guiding significance for older people individuals the most of worldwide, especially in East Asia, due to China's status as the country with the largest older population globally [25], and providing access to a substantial amount of data on napping [15]. This abundance of data enhances the representativeness and persuasiveness of research evaluating suitable sleep patterns and IC change about aging. Notably, in the context of Chinese sleep habits, the concept of taking a nap, particularly referring to the post-lunch rest [13,26], may be distinct from other countries' definitions of a nap as sleep at any time during the day [27].

In our study, excessive sleep duration was harmful to locomotion. Previous research has shown that engaging in physical activities is beneficial in reducing the risk of short sleep duration and improving sleep efficiency and quality [[28], [29], [30]]. Improving both general and leisure physical activities can be an effective way to prevent the decline in IC among older adults [7]. However, little information is available regarding the effects of prolonged sleep on physical function. Our findings suggest that there may be impaired locomotion when the nighttime and total sleep duration exceed 10 h. Interestingly, related studies indicate that higher sleep quality is independently associated with improved gait speed and reduced prevalence of falls mediating the effect of muscle strength in both men and women [31]. Our research evaluated locomotion also using kindred indicators such as walking speed, balance, and chair-stand tests, which further enriches the research findings on the correlation between sleep and locomotion in older people. This also indirectly proves the high consistency of IC's prediction of aging indicators.

Maintaining cognitive function is crucial for healthy aging, and assessing cognitive capacity plays a critical role in early identification and prevention of dementia [32]. Both insufficient and excessive sleep duration have been linked to cognitive and memory impairment, consistent with previous research findings [[33], [34], [35]]. Notably, naps of ≤60 min have shown significant cognitive benefits. Additionally, a study has demonstrated the positive effects of daytime napping on perceptual abilities, cognitive and psychomotor performance, as well as learning processes [36]. Therefore, incorporating scheduled daytime naps could be proposed as a potential solution to enhance the health and daily performance of older adults [26,27]. Furthermore, nighttime and total sleep duration <6 h may have adverse effects on psychological status, potentially exacerbating depressive and anxiety symptoms [37], particularly in females. However, it is worth noting that napping has been found to positively influence psychological capacity, providing a potential buffer and compensatory effect for sleep deprivation. Recent research indicates that high-quality lunchtime naps and meals indirectly enhance an individual's creativity through dual processes, including increased work engagement and reduced cognitive depletion [38].

We have observed an interesting phenomenon where napping is beneficial for psychological and cognitive function, but detrimental to athletic performance. Short to moderate naps provide a period of rest and rejuvenation, enhancing memory consolidation [39], alertness [40], and emotional regulation [41], thereby improving cognitive and psychological function. Conversely, prolonged or poorly timed naps may interfere with the quality and duration of nighttime sleep, leading to drowsiness and physical fatigue [42], which could negatively impact physical function and exercise, resulting in reduced locomotion [43].

In current research, the negative mediating effect of dyslipidemia suggests that appropriate daytime naps may improve IC change by retaining blood lipid stability. Previous research has adopted Linear Mendelian randomization analyses to demonstrate that genetically predicted 1-h longer sleep duration was associated with a 13% lower risk of metabolic syndrome, including dyslipidemia [44]. Our findings may explain that daytime napping could potentially serve as a compensatory mechanism for insufficient nighttime sleep and contribute to the regulation of stable blood lipid metabolism. In the Asian population, low BMI is strongly associated with an increased risk of mortality [45]. However, our current research did not find significant differences in BMI across different sleep duration groups, and no evidence was found to support BMI changes as a mediator in the relationship between sleep and IC outcomes. These results, particularly the absence of an effect of BMI on IC change, are surprising considering the known association between BMI and obstructive sleep apnea [46].

Within the study conducted among the older population in China, we propose a healthy sleep pattern for promoting healthy aging. One potential mechanism revealing a causal link between shortened sleep duration (less than 6 h) and mortality involves the induction of endothelial dysfunction resulting from reduced sleep, which progressively elevates cardiovascular risk [47]. In turn, further associations between cardiovascular risk and mortality outcomes are well known [14,26,27,48]. Meanwhile, individuals with prolonged sleep durations may harbor undiagnosed underlying diseases that trigger the release of proinflammatory markers, as both C-reactive protein and interleukin 6 have been correlated with extended sleep duration [49]. On the other hand, while daytime napping has demonstrated significant benefits for depression and cognitive performance, it could serve as a pragmatic response to daytime sleepiness caused by sleep deprivation, because late bedtimes and early wake-up times may disrupt circadian rhythms, leading to an extended duration of elevated melatonin levels upon waking, subsequently resulting in lengthier napping periods as a compensatory mechanism [50].

## 5 Strengths and limitations

This study has several strengths. Firstly, it presents a comprehensive summary and analysis of a large-scale prospective cohort comprised of middle-aged and older people individuals in China. This allows for the estimation of IC with a considerable degree of certainty, leveraging the habitual sleep pattern specific to the Chinese population. Moreover, the findings may serve as valuable references, particularly for the East Asian population. Secondly, meticulous adjustments were made to account for 29 significant confounding factors that seriously affect sleep. Thirdly, in the sensitivity analysis, individuals who utilized sedatives or had physical disabilities, zero total sleep time, or extreme IC values were excluded in order to mitigate the impact of reverse causality.

This study does entail several limitations. Self-reported questionnaires may overestimate sleep duration, particularly among individuals who sleep less [51]. It lacks information on night shift work and other potential factors that may influence sleep duration and IC. Further investigation is needed on factors such as personality traits and lifestyle. Residual confounding is also possible due to the lack of dietary information and other relevant confounders.

## 6 Conclusions

In this prospective cohort analysis, we found support that the most effective sleep regimen for maintaining IC level is to ensure a consistent duration of 6−8 h of sleep during the nighttime, complemented by ≤60 midday nap. This specific sleep pattern serves to actively facilitate the healthy process of aging, allowing for enhanced locomotion, and psychological and cognitive performance. Moreover, conducting prospective studies with objective exposure assessment on sleep patterns and longitudinal measurements on IC hold significant research value, particularly for populations residing in East Asia or those with a customary practice of midday napping.

## Contributors

Xing-Ling Chen contributed to the study concept and design, interpreting the data, and wrote and revised the manuscript. Jin Li and Shu-Ning Sun contributed to composing the statistical dataset, and performed the analyses. Xiao-Jiao Zhang and Jia-Hui Chen contributed to interpreting the data and critical revision of the manuscript. Ling-Jun Wang, Zhong-Qi Yang, and Shi-Hao Ni contributed to interpreting the data and critical revision of the manuscript. Lu Lu contributed to the study concept and design, interpreting the data and critical revision of the manuscript. All authors reviewed and approved the final version and no other person made a substantial contribution to the paper.

## Funding

This work was supported by a grant from the National Science Foundation of China (No. 82374406), the Young Elite Scientists Sponsorship Program by CAST (CACM-2021-QNRC2-B30), Guangdong Basic and Applied Basic Research Foundation (2023A1515030146), Guangzhou University of Chinese Medicine's Youth Elite Talents Cultivation "List Unveiling and Leadership" Team Project, and the Excellent Doctoral Dissertation Cultivation Project of the First Clinical School of Guangzhou University of Chinese Medicine in 2023 (YB202301).

## Ethical approval

CHARLS was a survey approved by the Ethical Review Committee of Peking University (approval number IRB00001052–11015), and the study data were anonymous. Each participant provided signed informed consent at the time of participation. There was no requirement for additional ethics approval for approved data users.

## Research data (data sharing and collaboration)

The data sets used and analyzed during the study are available online. Harmonized CHARLS. https://charls.charlsdata.com/pages/data/111/zh-cn.html

## Declaration of competing interest

The authors declare that they have no known competing financial interests or personal relationships that could have appeared to influence the work reported in this paper.
