# Supplementary material for: Validation of intrinsic capacity and healthy sleep pattern in middle-aged and older adults: a longitudinal Chinese study assessing healthy ageing
Source: J Nutr Health Aging. 2024 Sep 21;28(11):100365. doi: 10.1016/j.jnha.2024.100365 (PMC12879214; doi:10.1016/j.jnha.2024.100365)
Supplement: Supplementary file 1 [file mmc1.docx]

**Supplementary information**

**Methods**. Supplementary Methods

**Figure S1**. Flow diagram detailing the inclusion and exclusion criteria for participant selection in the study.

**Figure S2.** Confirmatory factor analysis (CFA) bifactor model for intrinsic capacity, illustrating the structural equation modeling used to assess intrinsic capacity with one general factor and five sub-factors.

**Figure S3.** Linear regression analysis of multi-variable adjusted association of three evaluation indicators of locomotion with sleep duration.

**Figure S4**. Linear regression analysis of multi-variable adjusted association of three evaluation indicators of sensory with sleep duration.

**Figure S5.** Linear regression analysis of multi-variable adjusted association of three evaluation indicators of vitality with sleep duration.

**Figure S6.** Linear regression analysis of multi-variable adjusted association of four evaluation indicators of cognitive capacity with sleep duration.

**Figure S7.** Restricted cubic spline models for the relationship between three evaluation indicators of locomotion and the sleep duration.

**Figure S8.** Restricted cubic spline models for the relationship between three evaluation indicators of sensory and the sleep duration.

**Figure S9.** Restricted cubic spline models for the relationship between three evaluation indicators of vitality and the sleep duration.

**Figure S10:** Restricted cubic spline models for the relationship between four evaluation indicators of cognitive capacity and the sleep duration.

**Figure S11:** Restricted cubic spline models for the relationship between IC change and nap/total sleep duration ratio.

**Figure S12.** Subgroup analyses for the association between the total score of intrinsic capacity and less total sleep duration by gender, age, residence, BMI and median 2011 IC.

**Figure S13**. Subgroup analyses for the association between the total score of intrinsic capacity and over total sleep duration by gender, age, residence, BMI and median 2011 IC.

**Figure S14.** Subgroup analyses for the association between the total score of intrinsic capacity and less nighttime sleep duration by gender, age, residence, BMI and median 2011 IC.

**Figure S15**. Subgroup analyses for the association between the total score of intrinsic capacity and over nighttime sleep duration by gender, age, residence, BMI and median 2011 IC.

**Figure S16.** Subgroup analyses for the association between the total score of intrinsic capacity and moderate nap duration by gender, age, residence, BMI and median 2011 IC.

**Figure S17** Subgroup analyses for the association between the total score of intrinsic capacity and over nap duration by gender, age, residence, BMI and median 2011 IC.

**Figure S18**. Subgroup analyses for the association between the intrinsic capacity and less/over nap duration by nighttime sleep duration.

**Table S1.** Baseline sample characteristics according to sleep duration categories.

**Table S2.** Sensitivity analysis: model 1 was used to evaluate the original IC total score of the middle-aged and elderly population's sleep duration in 2011.

**Table S3.** Sensitivity analysis: model 2 was used to evaluate the relationship between IC and sleep duration in the elderly population after excluding the use of sedatives or sleeping pills.

**Table S4.** Sensitivity analysis: model 3 was used to evaluate the relationship between IC and sleep duration in the elderly population after excluding the respondents with physical disabilities.

**Table S5.** Sensitivity analysis: model 4 was used to evaluate the relationship between IC and sleep duration in the elderly population after excluding the total sleep duration value of zero.

**Table S6.** Sensitivity analysis: model 5 was used to evaluate the relationship between IC and sleep duration in the elderly population after excluding the extreme IC total score.

**Table S7.** Sensitivity analysis: model 5 was used to evaluate the relationship between IC and sleep duration in the elderly population who completed receding position procedures.

**Table S8.** The Mediation analysis of 14 chronic diseases and BMI changes between IC and total sleep duration less than 6 hours.

**Table S9.** The Mediation analysis of 14 chronic diseases and BMI changes between IC and total sleep duration more than 10 hours.

**Table S10.** The Mediation analysis of 14 chronic diseases and BMI changes between IC and moderate nap duration less than 60 minutes.

**Table S11.** The Mediation analysis of 14 chronic diseases and BMI changes between IC and nap duration more than 60 minutes.

**Table S12.** The Mediation analysis of 14 chronic diseases and BMI changes between IC and nighttime sleep duration less than 6 hours.

**Table S13.** The Mediation analysis of 14 chronic diseases and BMI changes between IC and nighttime sleep duration more than 10 hours.

**Methods. Supplementary Methods**

**Data and study participants**

We used publicly available de-identified data from the China Health and Retirement Longitudinal Study (CHARLS) to measure the IC construct and to examine daytime, nighttime, and overall sleep duration within it. The CHARLS is a nationally representative longitudinal survey of Chinese residents aged 45 and older, which aims to provide high-quality data for scientific research on the health, economic, and social well-being of the older people population. The baseline national wave of CHARLS was fielded in 2011 and included about 150 counties/districts and 450 villages/resident committees. All respondents from the five waves (2011-2020 years) were invited to participate in the Life History Survey, which included a series of questions about background, health status and behaviors, health care utilization and expenditure, income and consumption, family and social networks, and psychological well-being. The survey also includes physical examinations and biomarker measurements for a subsample of participants([1](#_ENREF_1)).

We established a cohort consisting of individuals who, at their initial follow-up, met the following inclusion criteria: (1) initial age between 45 and 100, (2) completion of at least one sleep questionnaire, (3) at least one follow-up record, and (4) exclusion of participants with more than 40% missing data in the evaluation of IC. The multiple imputation method was utilized to impute the missing data, thereby reducing bias and enhancing the efficiency of our analyses. A flowchart detailing participant inclusion and exclusion criteria is provided in Figure S1.

It is worth noting that only the first (2011) and third (2015) waves have published data on physical examinations and blood testing, and changes in IC are calculated only for these two years. Therefore, we only considered the changes in IC over these four years and did not use Mixed-Effects (Multilevel) Models for data processing.

**Exposure assessment**

As it is not feasible to accurately measure the actual sleep time using objective methods like polysomnography in large studies, sleep information was recorded by asking the following standardized questions:

(a) During the past month, how many hours of actual sleep did you get at night (average hours for one night)? (This may be shorter than the number of hours you spend in bed);

(b) During the past month, how long did you take a nap after lunch?

The estimated nighttime sleep time was defined as less than the time interval between bedtime and wake-up time, and napping duration was estimated by self-reported daytime sleep time. The estimated total daily sleep length was the sum of these two parameters. To avoid bias caused by a single questionnaire on exposure factors, we used the average of sleep information from 2011 and 2013. We specifically analyzed the length of nighttime sleep and post-meal naps as critical components of the sleep pattern. Recognizing that napping can affect nighttime sleep, we also calculated the ratio of daytime nap duration to nighttime sleep duration to better understand their combined impact on IC. Additionally, we compared populations that adhere to this sleep mode with those who do not, to uncover potential differences in their functional abilities and IC changes.

**Intrinsic capacity measurement**

The predictive value of IC in this cohort study has been established in previous research by a structural equation model (SEM)([2](#_ENREF_2), [3](#_ENREF_3)). IC encompasses five distinct subdomains, including locomotion, sensory, vitality, psychological capacity, and cognitive capacity. Each subdomain is evaluated using various indicators, such as 1) locomotion: balance, walking speed time, and chair-stand test; 2) sensory: vision and hearing impairments; 3) vitality: forced expiratory volume, grip strength, and hemoglobin; 4) psychological capacity: ten-item Center for Epidemiological Studies-Depression (CES-D) scale (To avoid misleading correction, we did not include sleep length and sleep quality in the assessment); 5) cognitive capacity: immediate and delayed recall, drawing and math. Please refer to Supplementary Materials for specific measurement methods.

The Structural Equation Model (SEM) included a confirmatory factor analysis (CFA) based on sub-factors identified from a previous exploratory factor analysis (EFA) using the same indicators. A bi-factor model, comprising one general factor and five sub-factors, was fitted and is presented in Figure S2. The goodness of fit statistics, such as the Root Mean Square Error of Approximation (RMSEA), Comparative Fit Index (CFI), and Tucker-Lewis Index (TLI), were used to assess the model fit. Acceptable values for CFI and TLI are typically close to or greater than 0.85, with values above 0.90 considered indicative of an excellent fit.

The fit statistics indicated a good fit of the model to the data (RMSEA = 0.094, CFI = 0.898, TLI = 0.843). The IC score was standardized by subtracting the mean and dividing by the standard deviation, where a higher score indicates a stronger capacity to perform valued activities. The IC score was treated as a continuous variable in our analysis.

**Covariates**

The covariates include the basic demographic characteristics, lifestyle behaviors, socioeconomic status, and health status of the population collected in 2011. These covariates were included to control for potential confounding factors that could influence the relationship between sleep patterns and IC, ensuring that the observed associations are as accurate as possible.

Demographic variables were collected, which included age (years), gender (male/female), residence (rural/urban), educational level (Primary school or lower / Middle school / High school or higher), and married status (current or former/never married). Socioeconomic status specifically refers to household annual income. Lifestyle behaviors were obtained from a self-report questionnaire that included the physical activity level (MET-PA) (vigorous/moderate/leisure activities), smoking status (current or former/never smoker), drinking status(drinker/nondrinker), memory(good/fair/poor), mental health assessment using the CES-D scale, and taking tranquilizers or sleeping pills(yes/no). For sleep quality, participants were asked to report the frequency of restless sleep during the last week with four categorized answers (good/fair/poor). Health conditions contained physical disabilities (yes/no) and 14 medically diagnosed conditions (yes/no), including history of hypertension, dyslipidemia, diabetes, cancer, chronic lung diseases, liver disease, heart diseases, stroke, kidney diseases, digestive diseases, psychiatric problems, memory-related diseases, arthritis or rheumatism, and asthma. CHARLS medical physical examinations recorded body mass index BMI, calculated as measured weight (kg) divided by the square of height (m^2^).

**Descriptive statistics**

Based on the recent consensus reached by the American Academy of Sleep Medicine (AASM) and the Sleep Research Society (SRS), it is recommended that adults have the best sleep time of 7 hours or more per night([4](#_ENREF_4)). We take 7 h as the median and 6-8 h of sleep time as a control reference. We categorized the estimated nighttime and total daily sleep duration into four groups (<6 h, 6–8 h which was used as the reference, 8–10 h, and >10h), and categorized the estimated napping time into three groups (0 minute, which was used as the reference, ≤60 mins, >60 mins). Continuous variables were expressed as mean (standard deviation), while categorical variables were presented as numbers (percentages). One-way analysis of variance (ANOVA) was utilized to compare the mean values of continuous variables, while the chi-square test was employed to analyze the percentage distribution of categorical variables. We conducted cubic spline analysis using the "rcs" package, and subgroup analysis was performed using the "Publish" package. The mediation effect was assessed using the "mediation" package in R version 3.6.1. The two-sample Mendelian randomization (MR) analysis was performed using the R package “TwoSampleMR”. Statistical significance was determined based on a two-sided *P* < 0.05.

**Linear and restricted cubic spline regression**

Multivariable linear regression models were used to evaluate the association of daytime, nighttime, and overall sleep patterns with IC, adjusting for current health conditions, socioeconomic status, demographic characteristics, and lifestyle information. The observed correlation is represented by the marginal effects, which refer to the change in the dependent variable (IC) resulting from a one-unit change in an independent variable (such as sleep duration or nap time) while holding other variables constant. Marginal effects (ME) and their corresponding 95% confidence intervals (CIs) were determined for IC changes within its five subdomains. Our primary approach involved utilizing the comprehensive information available in the survey data to account for as many potential confounding factors as possible. This is crucial because the estimated relationship between IC and beneficial factors may be distorted when the outcome variable is influenced by associations with the error term([5](#_ENREF_5)). Restricted cubic splines were used to explore the shape of the association between estimated daily sleep pattern and the outcomes (created by SAS LGTPHCURV9 Macro).

**Subgroup and sensitivity analyses**

To thoroughly and reliably investigate the relationship between sleep pattern and IC, we conducted subgroup analysis and sensitivity analysis to validate our findings. To facilitate a clearer comparison, we will categorize the study participants into six distinct groups based on their sleep duration: individuals with total sleep time >10 h and <6 h, those with night sleep time >10 h and <6 h, participants engaging in napping ≤60 mins and >60 mins. This approach will allow for a more nuanced analysis of the effects of varying sleep patterns on the observed outcomes.

For the subgroups, (a) examined whether the effects of sleep pattern on events were consistent across different age groups (<60 years old / ≥60 years old), considering that the need for sleep may increase with age. (b) categorized overweight as a BMI of 24 greater than or equal to 24 but less than 30, general obesity as a BMI of 30 or higher, in accordance with the World Health Organization's definition for international comparability. Thus, the data into BMI (normal / overweight / obesity) were stratified to explore the specific impact of IC change on obesity, as obesity, defined as a BMI of ≥30kg/m^2^, has been shown to be strongly associated with sleep apnea([6](#_ENREF_6)). (c) stratified the data according to residence (rural / urban areas) to investigate the impact of sleep habits on IC change, as there is a significant difference in lifestyle habits between these areas in developing countries. (d) performed a gender-stratified analysis (male/female) and the stratification of the median of IC in 2011 (above median/under median). (e) subgroup analyses for the association between the intrinsic capacity and less/over nap duration by nighttime sleep duration.

In the sensitivity analysis, several steps were taken. Firstly, we excluded participants who reported using sedatives or sleeping pills. Moreover, respondents with physical disabilities, who spend longer periods of time in bed due to long-term paralysis, were eliminated. Additionally, we also removed data points with extreme values of IC (add or subtract five times the standard deviation). Also, we excluded respondents those who were not retired. Finally, to avoid occasional extreme insomnia data, data points with a total sleep length value of zero were ruled out.

**Mediation effect analysis**

To examine the potential mediation of various factors on the influence of IC and its five subdomains, our study investigated the mediating effects of changes in BMI and the occurrence of chronic diseases (refers to the onset of chronic disease that was diagnosed in 2013 or 2015 but not in 2011) on the associations between daytime, nighttime, all-day sleep pattern, and IC change. The grouping method is consistent with subgroup analysis and sensitivity analysis. The mediation analysis encompassed several procedures: (a) computed the linear relationship between BMI changes, chronic diseases, and IC. (b) a linear model was utilized to analyze the results, encompassing all variables pertaining to sleep pattern. (c) employed the mediation ratio determination methods outlined by Preacher, Kristopher J, and Hayes, Andrew F([7](#_ENREF_7)). We established a 95% confidence interval using the bootstrap method and repeated the procedure 5000 times.

**Intrinsic capacity data obtained from CHARLS**

**Walking speed time:** In CHARLS, each participant aged 60 and above was eligible for the timed walk test. In addition, prior to the actual test, participants were asked if they had any problems from recent surgery, injury, or other health conditions that might prevent them from walking. Only persons aged at least 60 years, willing to do the test, and able to walk (walking aids were permitted) were asked to walk 2.5 meters at their usual walking pace, twice. The time for both walks was recorded separately. In our analysis, we use the time (seconds) of the two trials.

**Chair-stand test:** The chair-stand test was used in CHARLS as a measure of physical performance, assessed the time required to rise from a chair to a full standing position five times with arms folded across the chest, with slower times reflecting worse function. The test incorporated the use of the respondent’s own armless, straight-backed chair. The time taken for the full stand was recorded in seconds. Participants were considered ineligible if they could not stand up without assistance; the use of walking aids, such as a walker or cane, was not permitted. The test was stopped if the person became too tired or short of breath, if the person used their hands, or if the nurse assessor felt concerned for the person’s safety.

**Balance:** Static balance was evaluated in CHARLS through three separate tests, which formed part of the Short Physical Performance Battery 1. Participants were ineligible for the tests if they were chair-bound or wheelchair-based; if it became clear after discussion that they were too unsteady on their feet; if they found it painful to stand; or if either the nurse assessor or the participant, considered the test unsafe. We included data obtained for three components of the balance test: semi-tandem stand, full-tandem stand, and side-by-side stand: (a) Semi-tandem stand: Participants had to stand with the side of the heel of one foot touching the big toe of the other foot for at least 10 s. Participants unable to hold the position for 10 s scored one and no further tests were attempted. Those able to hold the position for 10 s moved on to the full-tandem stand; (b) Full-tandem stand: For this test, participants had to stand with the heel of one foot in front of and touching the toes of the other foot. Those aged 70 and above and able to hold the position for at least 30 seconds scored two points for this test; those aged below 70 and able to hold the position for at least 60 seconds scored two points for this test; those able to hold the position less than required time for certain age scored one point for this test; Those unable to hold this position scored no additional points; (c) Side-by-side stand: Participants were asked to stand with feet together, side-by-side, for at least 10 seconds, using their arms, bending their knees or moving their body to maintain balance, but not moving their feet. If the participant was unable to hold the position for 10 s, a score of zero was recorded and no further tests were attempted. Those able to hold the position for 10 seconds moved on to the semi-tandem stand. Note that semi-tandem stand and full-tandem stand are comparably more difficult than side-by-side stand, and in CHARLS 2011, a side-by-side stand was tested after the semi-tandem stand and full-tandem stand, with a low response rate. Therefore, we imputed side-by-side stand as one, if the participant was able to complete the semi-tandem stand and full-tandem stand; those unable to complete this test scored no additional points.

Grip strength: The grip strength test was used in CHARLS to test upper body strength. Handgrip strength (kg) of the dominant hand was assessed using a hand-held dynamometer. In the CHARLS 2011 wave, there were two measures for each hand. An average of the dominant hand was calculated for analysis (if both hands were reported as the dominant hand, we chose the larger measure). Any measurements carried out incorrectly or participants refused to perform the test were not included.

**Forced expiratory volume (FEV):** Lung function was measured in CHARLS using a spirometer. Eligible participants were asked to stand or sit, take a deep breath, and blow into the spirometer as hard as they could. They were then required to repeat the procedure to give three technically satisfactory blows. The highest technically satisfactory measure of FEV was used in the analysis.

**Blood assay:** The CHARLS wave 2011 collected venous blood samples, and a complete blood count analysis was undertaken at local CDC laboratories (this included hemoglobin, hematocrit, white blood cell count, platelet counts, and mean corpuscular volume). Three tubes of venous blood were collected from each participant by medically trained staff from the China CDC, using a standard protocol. Detailed information on the technicalities of the blood analysis, the internal quality control, and the external quality assessment for the laboratory have been described on the website of CHARLS (http://charls.pku.edu.cn/index/en.html).We used hemoglobin in this study for validation analysis.

**Sensory:** Hearing and vision impairments were measured in CHARLS using self-report. Hearing status was assessed by asking participants to rate their hearing (using a hearing aid if they used one) as excellent, very good, good, fair, or poor. For vision, participants were also asked ‘How good is your eyesight for seeing things at a distance, like recognizing a friend across the street (with glasses or corrective lenses if you wear them)’ and ‘How good is your eyesight for seeing things up close, like reading ordinary newspaper print’. Response options (excellent/very good/good/fair–poor) were categorized as above.

**Cognition:** The CHARLS data include scores on two tests of cognitive function: episodic memory and intact mental status 2,3. Memory recall data were based on the participant’s ability to recall the same list of words four minutes later (delayed recall). Episodic memory measures were obtained from delayed recall scores. Another cognitive measure was based on some components of the mental status questions of the Telephone Interview of Cognitive Status (TICS) battery 2, including the following: Serial 7 test, which requires the person to subtract 7 from 100 (up to five times); assessment of the person’s need of explanation, or use of an aid such as paper and pencil to undertake the serial 7 test; ability to accurately identify the current date (month, day and year) and season; and ability to redraw a picture shown to the person.

**Affect:** Affect was assessed using the ten-item Center for Epidemiological Studies-Depression (CES-D) scale4, except for the sleep dimension which was considered independently (see the following measure). Six of the nine CES-D items (i.e., was bothered, felt hopeful, felt depressed, was happy, felt lonely, felt fearful) were considered to be depressed mood items, while the remaining three (i.e., everything was an effort, had trouble keeping mind, and could not get going) were regarded as somatic complaints items. In order to allow comparison with Beard et al.’ s 5 previous validation methodology, nine items collected in CHARLS referring to how the participant felt and behaved during the last week. Each item corresponded with four categorized answers, rarely or none of the time (<1 day), some or a little of the time (1-2 days), occasionally or a moderate amount of the time (3−4 days), and most or all of the time (5−7 days). A summary CES-D score was derived by adding responses to all nine categorized questions.

**References**

1. Zhao Y, Hu Y, Smith JP, Strauss J, Yang G. Cohort profile: the China Health and Retirement Longitudinal Study (CHARLS). International journal of epidemiology. 2014;43(1):61-8.

2. Beard JR, Si Y, Liu Z, Chenoweth L, Hanewald K. Intrinsic Capacity: Validation of a New WHO Concept for Healthy Aging in a Longitudinal Chinese Study. The journals of gerontology Series A, Biological sciences and medical sciences. 2022;77(1):94-100.

3. Beard JR, Officer A, de Carvalho IA, Sadana R, Pot AM, Michel JP, et al. The World report on ageing and health: a policy framework for healthy ageing. Lancet (London, England). 2016;387(10033):2145-54.

4. Panel: CC, Watson NF, Badr MS, Belenky G, Bliwise DL, Buxton OM, et al. Joint Consensus Statement of the American Academy of Sleep Medicine and Sleep Research Society on the Recommended Amount of Sleep for a Healthy Adult: Methodology and Discussion. Journal of Clinical Sleep Medicine. 2015;11(08):931-52.

5. Violato M, Petrou S, Gray R, Redshaw M. Family income and child cognitive and behavioural development in the United Kingdom: does money matter? Health economics. 2011;20(10):1201-25.

6. Romero-Corral A, Caples SM, Lopez-Jimenez F, Somers VK. Interactions between obesity and obstructive sleep apnea: implications for treatment. Chest. 2010;137(3):711-9.

7. Preacher KJ, Hayes AF. SPSS and SAS procedures for estimating indirect effects in simple mediation models. Behavior research methods, instruments, & computers : a journal of the Psychonomic Society, Inc. 2004;36(4):717-31.

8. Guralnik JM, Simonsick EM, Ferrucci L, et al. A short physical performance battery assessing lower extremity function: association with self-reported disability and prediction of mortality and nursing home admission. J Gerontol.

1994;49(2):M85-M94.

9. Lei X, Hu Y, McArdle JJ, Smith JP, Zhao Y. Gender differences in cognition among older adults in China. J Hum Resour.2012;47(4):951-971.

10. Lei X, Smith JP, Sun X, Zhao Y. Gender differences in cognition in China and reasons for change over time: evidence from CHARLS. J Econ Ageing. 2014;4:46-55.

11. Carleton RN, Thibodeau MA, Teale MJ, et al. The center for epidemiologic studies depression scale: a review with a

theoretical and empirical examination of item content and factor structure. PloS One. 2013;8(3):e58067.

12. Beard JR, Jotheeswaran AT, Cesari M, de Carvalho IA. The structure and predictive value of intrinsic capacity in a longitudinal study of ageing. BMJ Open. 2019;9(11).

**
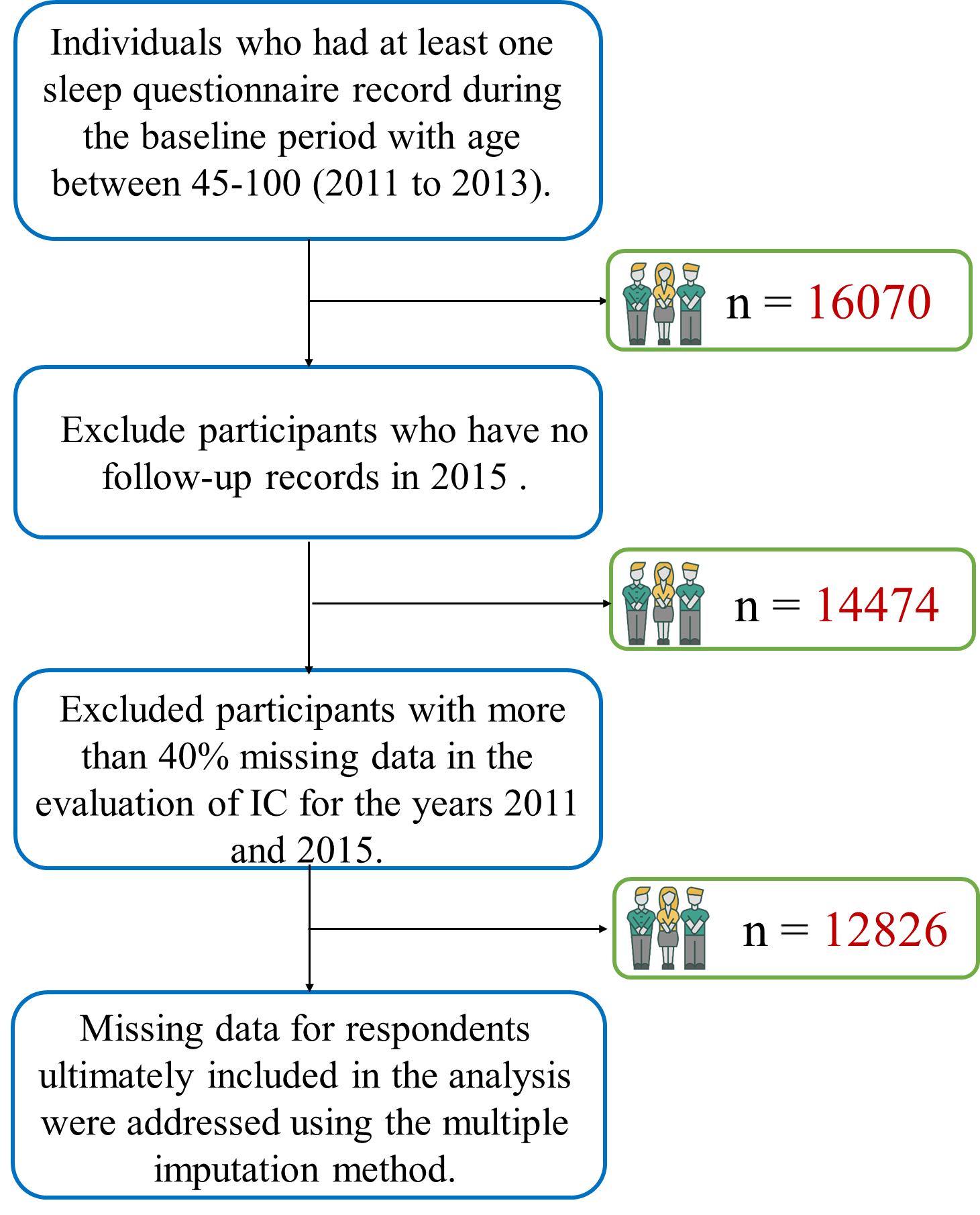
**

**Figure S1:** Flow diagram detailing the inclusion and exclusion criteria for participant selection in the study.

**Figure S2**. Bifactor CFA model of intrinsic capacity. CFA, confirmatory factor analysis.


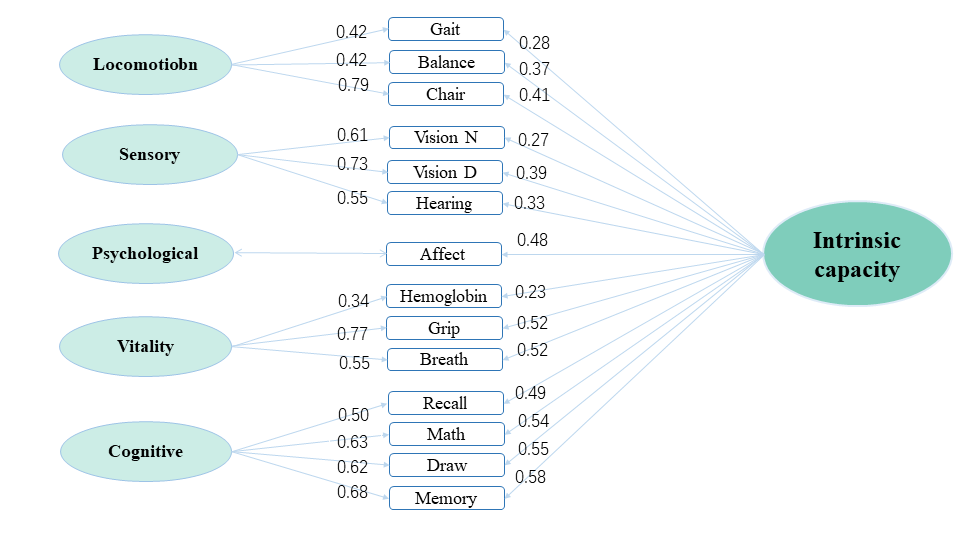


Figure S3. Linear regression analysis of multi-variable adjusted association of three evaluation indicators of locomotion with sleep duration.


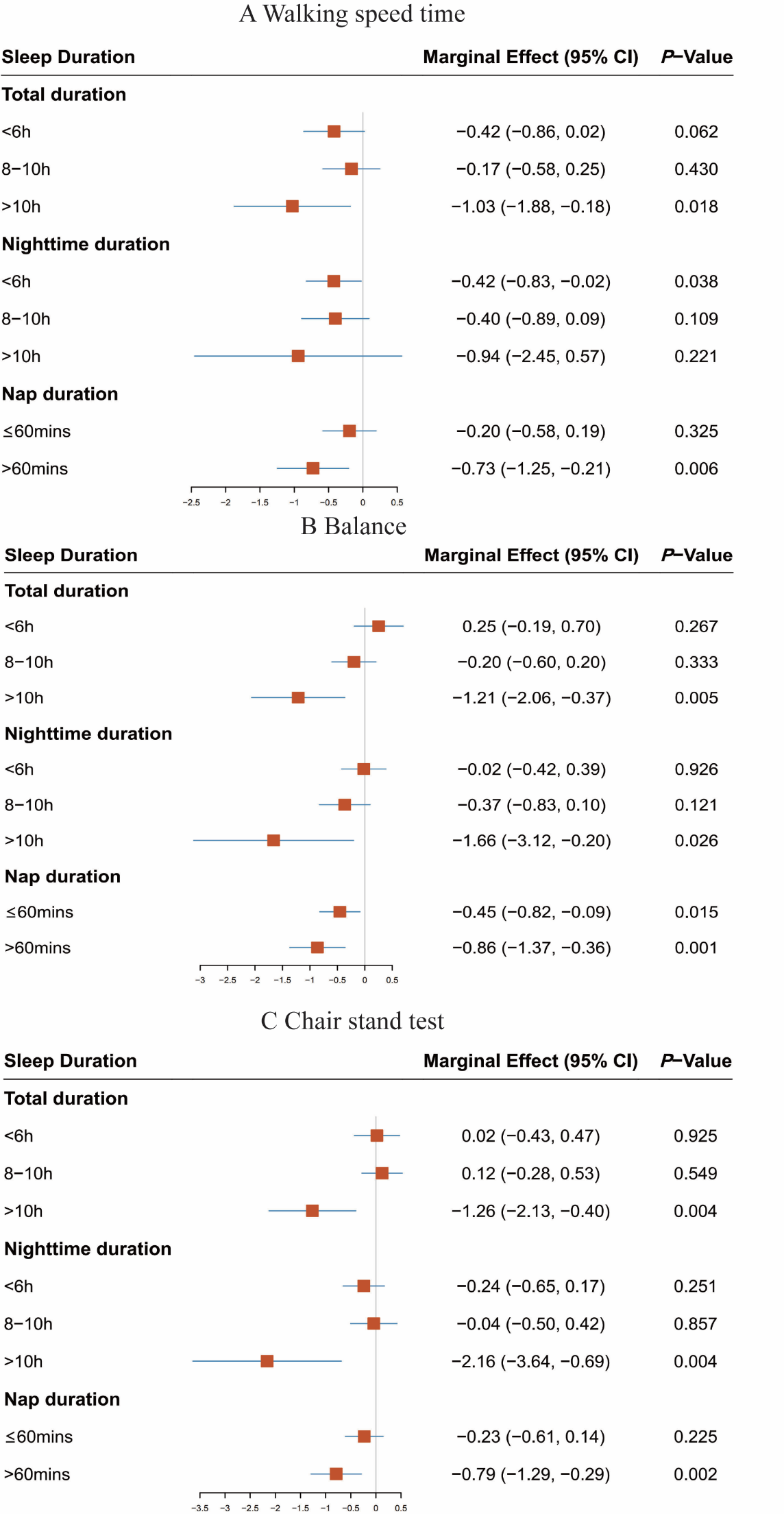


Figure S4. Linear regression analysis of multi-variable adjusted association of three evaluation indicators of sensory with sleep duration.


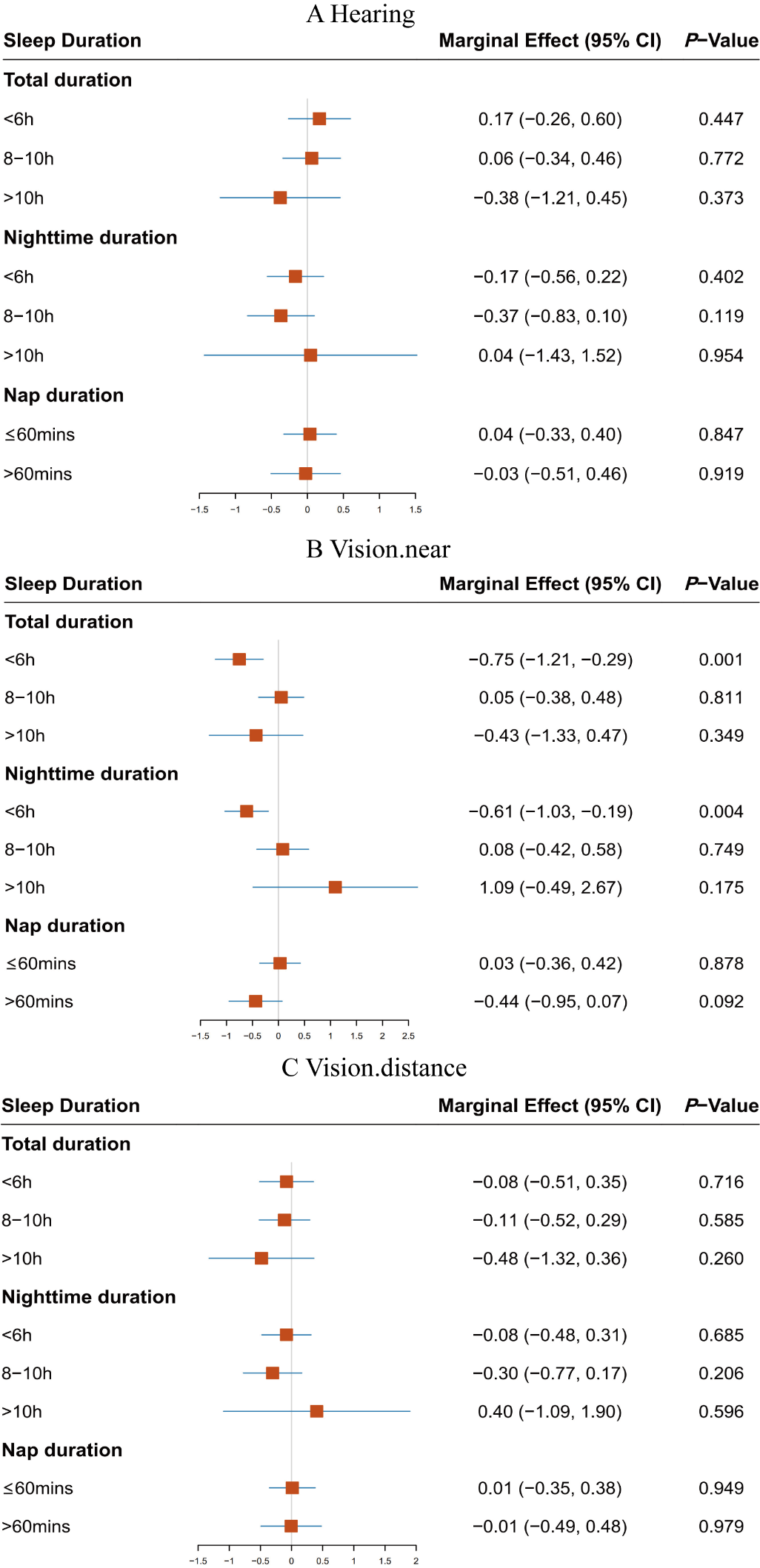


Figure S5. Linear regression analysis of multi-variable adjusted association of three evaluation indicators of vitality with sleep duration.


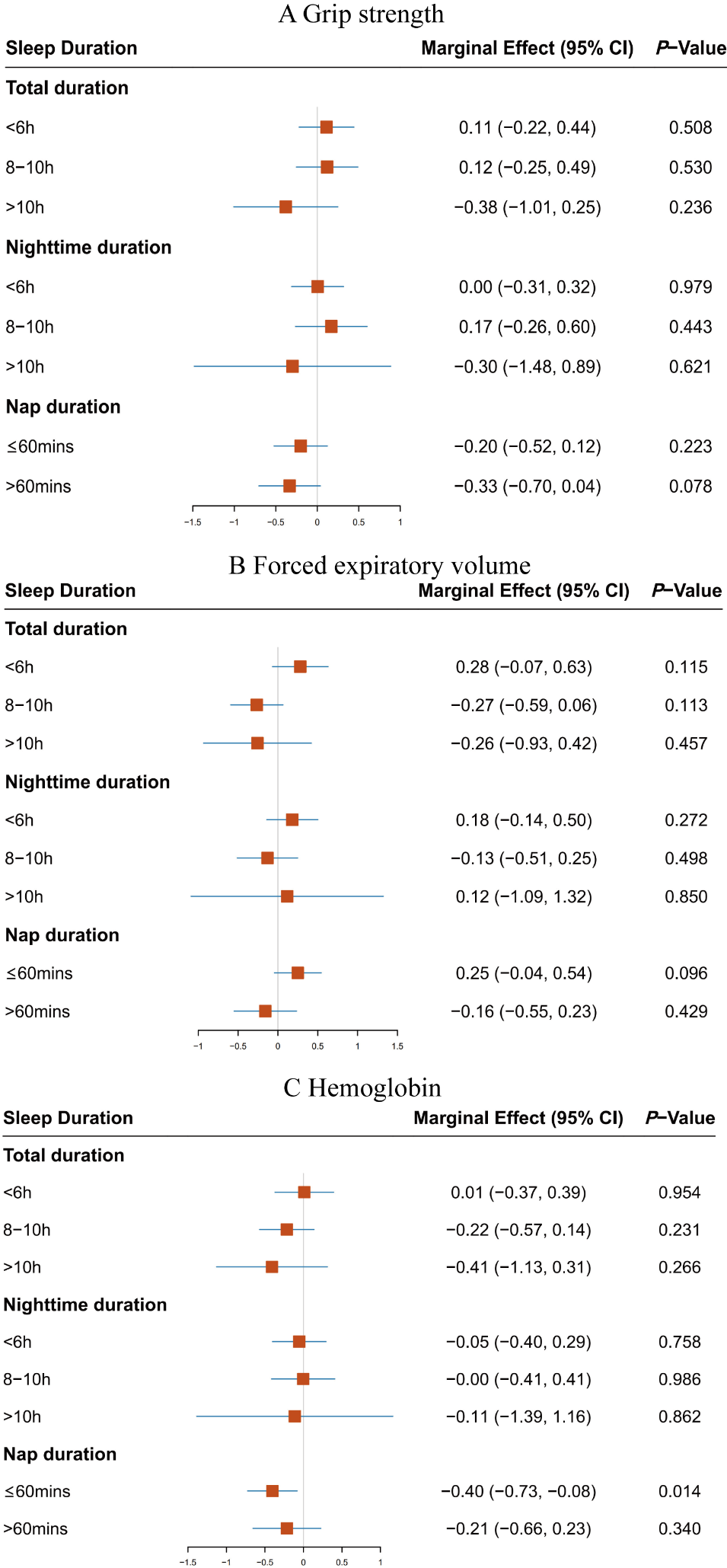


Figure S6. Linear regression analysis of multi-variable adjusted association of four evaluation indicators of cognitive capacity with sleep duration.


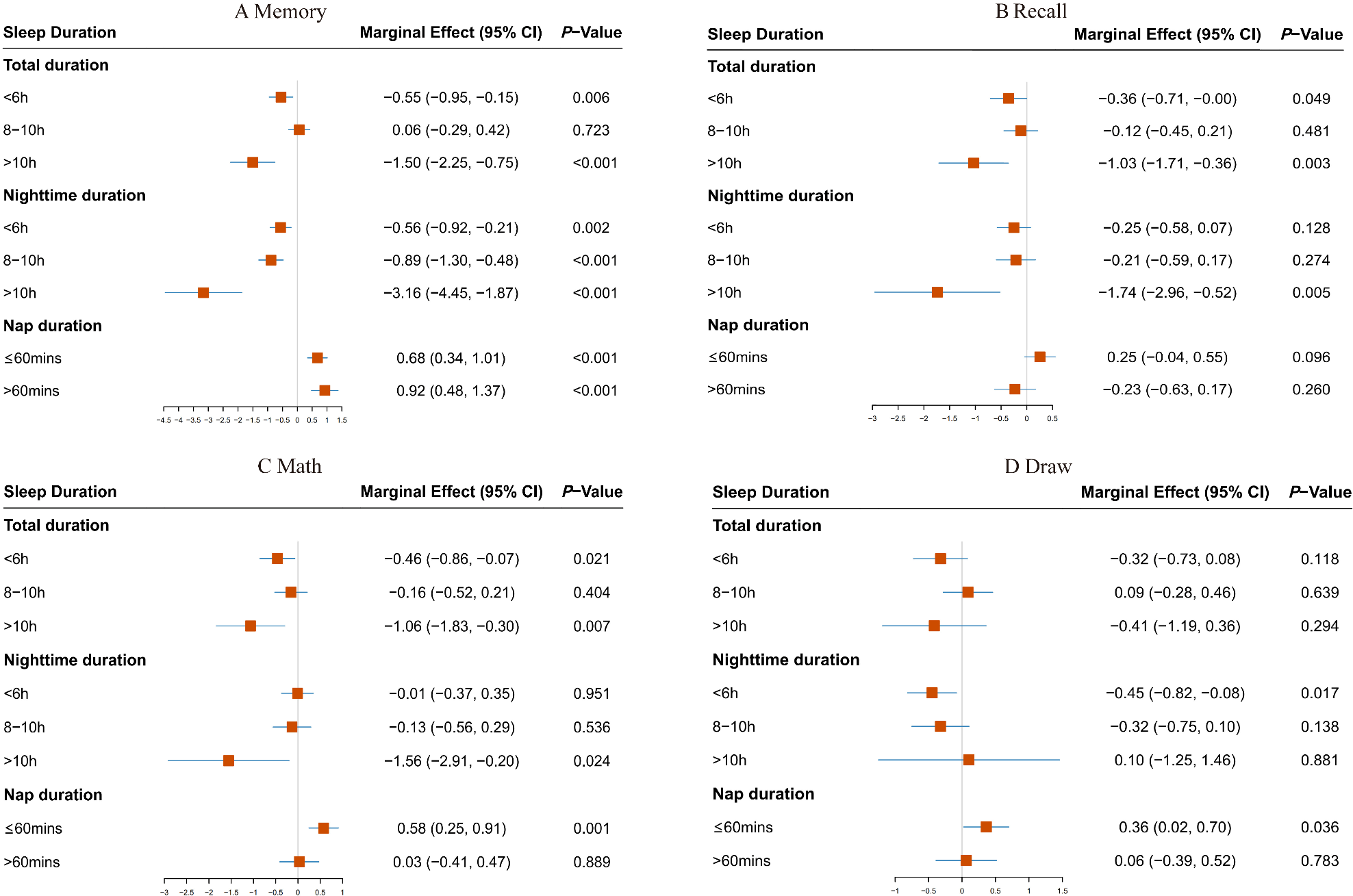


Figure S7: Restricted cubic spline models for the relationship between three evaluation indicators of locomotion and the sleep duration in the CHARLS (2011–2015).

A Total Sleep Duration


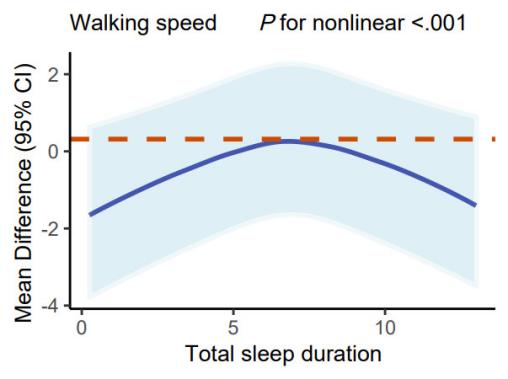

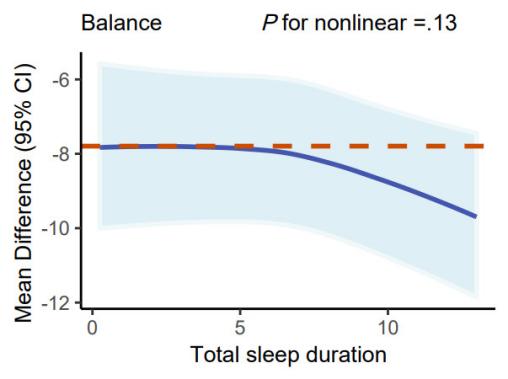

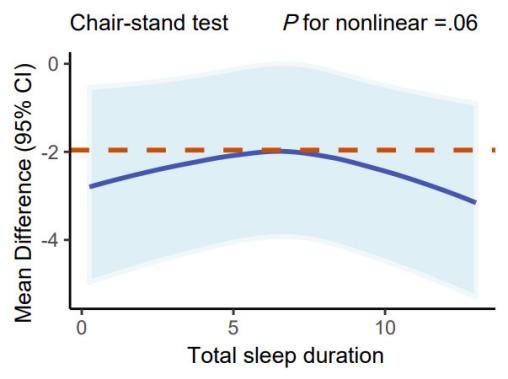


B Nighttime Sleep Duration


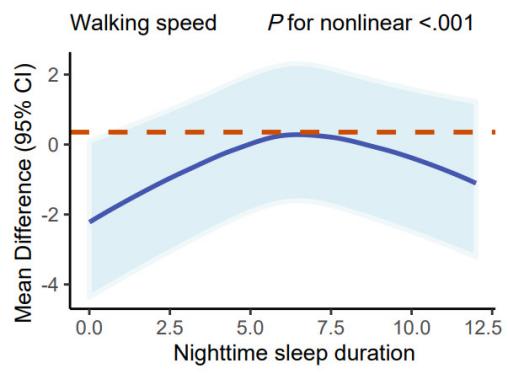

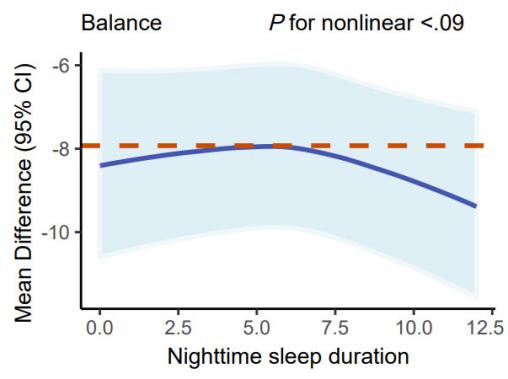

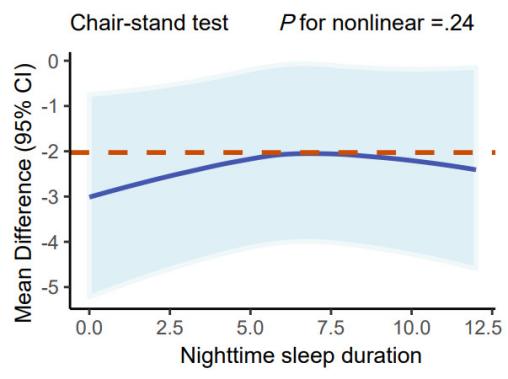


C Nap Duration


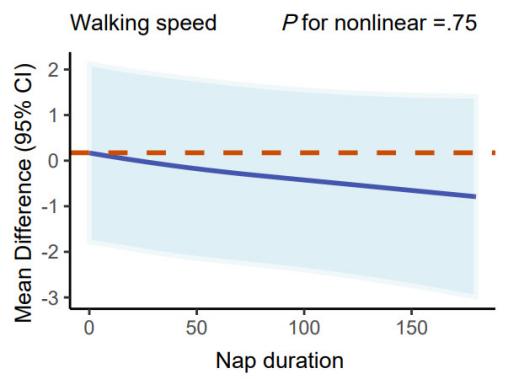

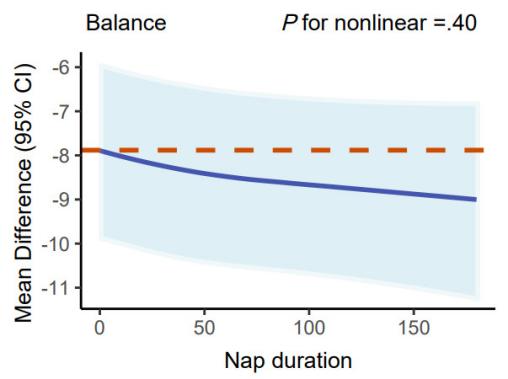

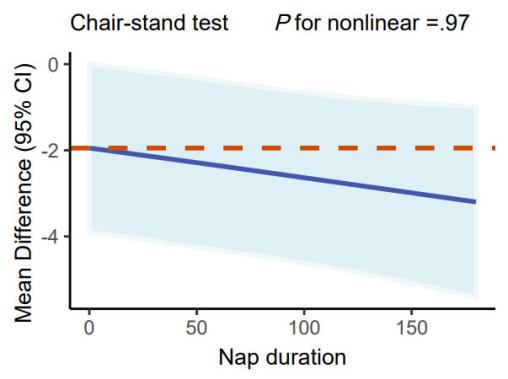


Figure S8: Restricted cubic spline models for the relationship between three evaluation indicators of sensory and the sleep duration.

A Total Sleep Duration


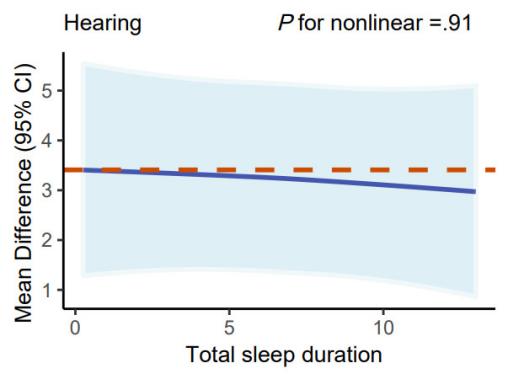

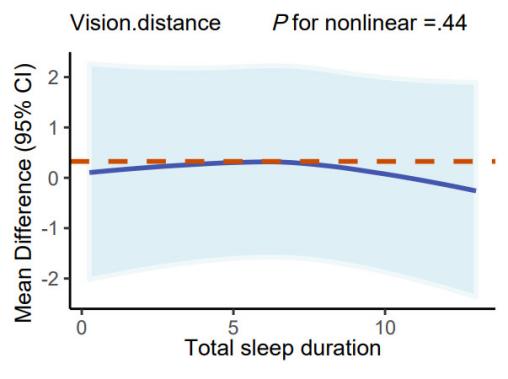

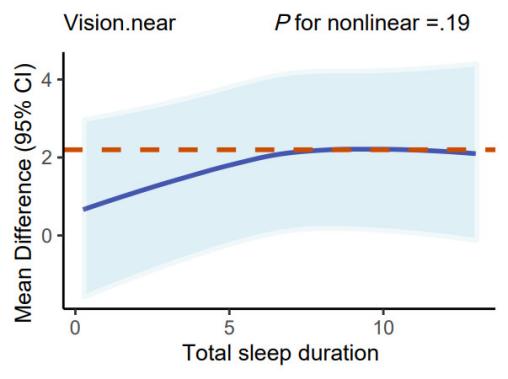


B Nighttime Sleep Duration


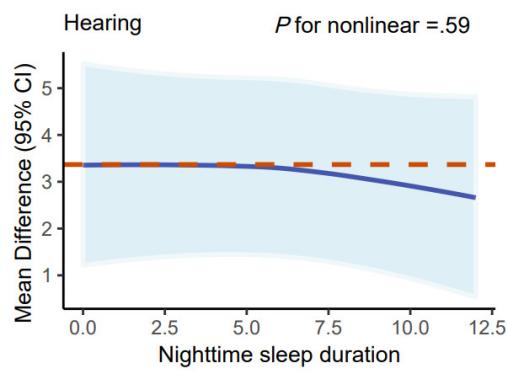

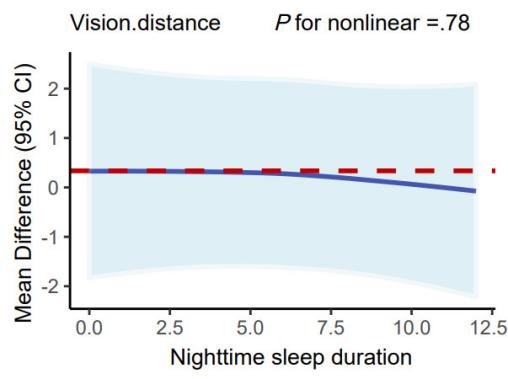

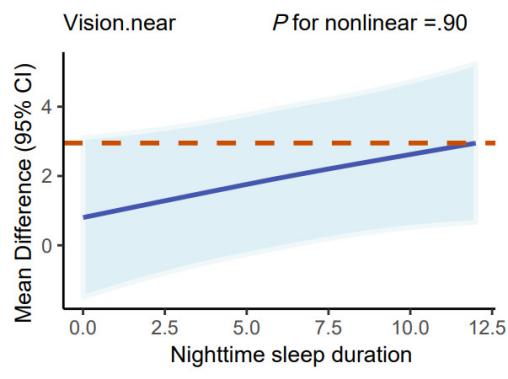


C Nap Duration


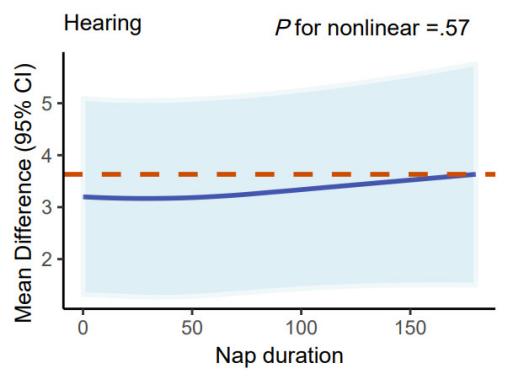

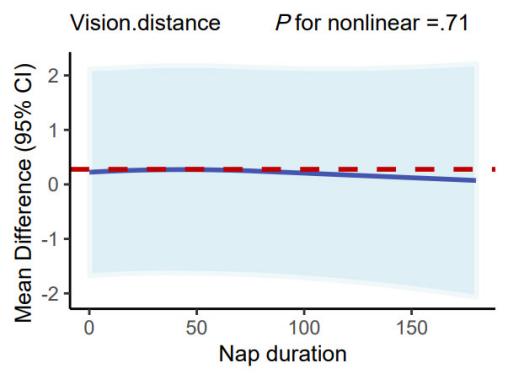

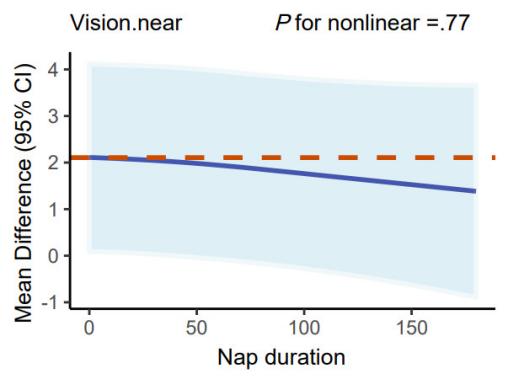


Figure S9: Restricted cubic spline models for the relationship between three evaluation indicators of vitality and the sleep duration.

A Total Sleep Duration


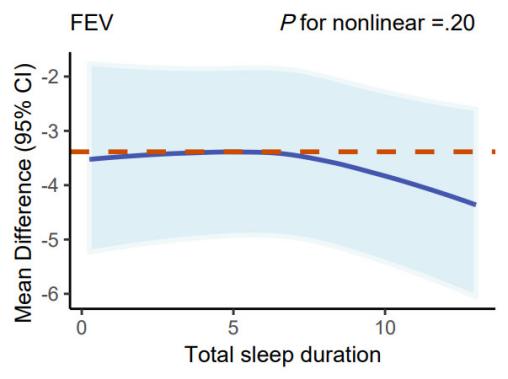

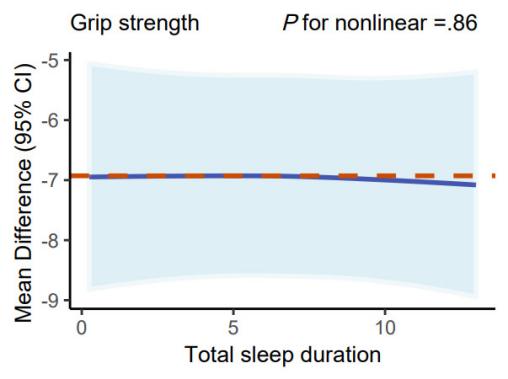

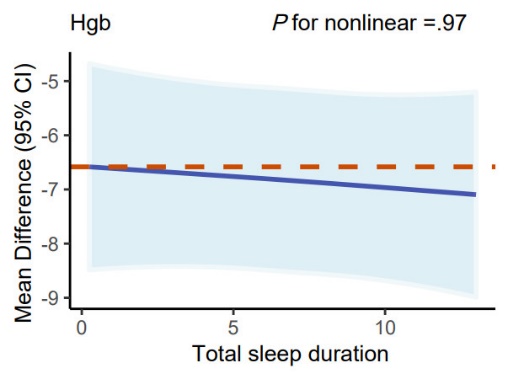


B Nighttime Sleep Duration


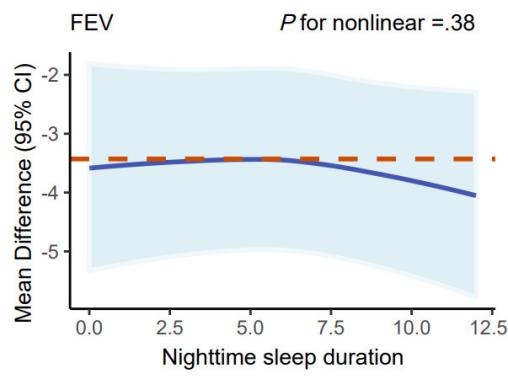

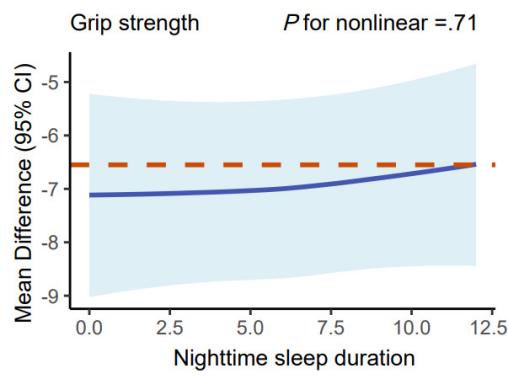

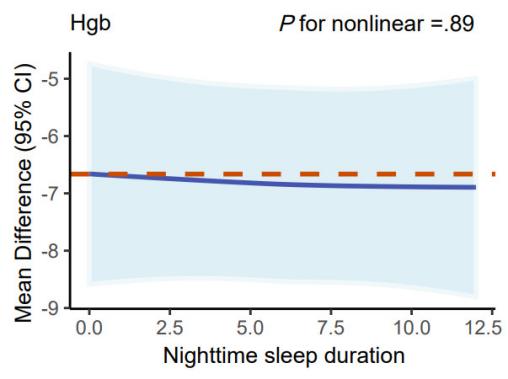


C Nap Duration


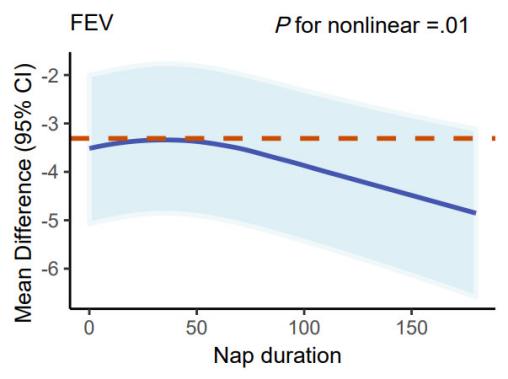

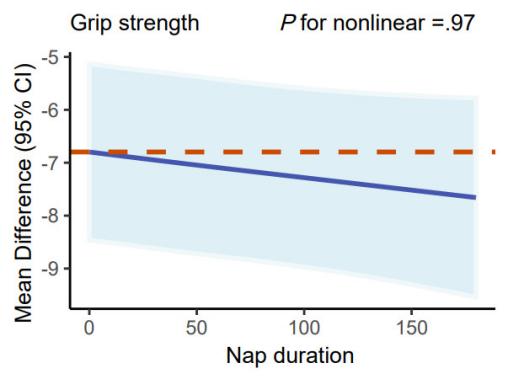

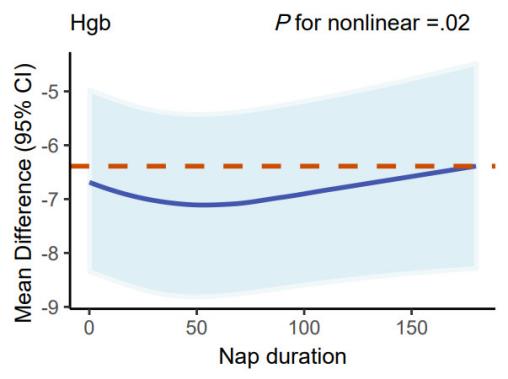


Figure S10: Restricted cubic spline models for the relationship between four evaluation indicators of cognitive and sleep duration.

A Total Sleep Duration


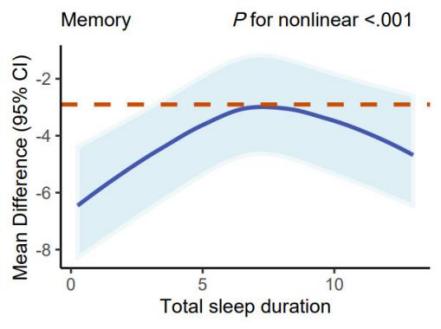

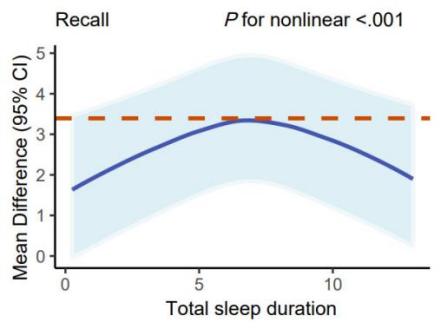


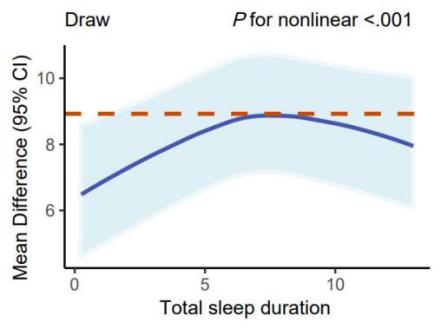

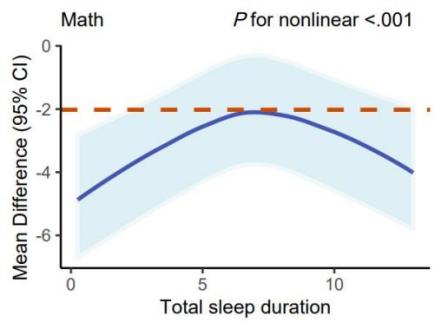


B Nighttime Sleep Duration


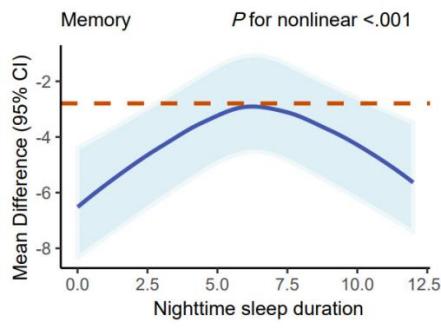

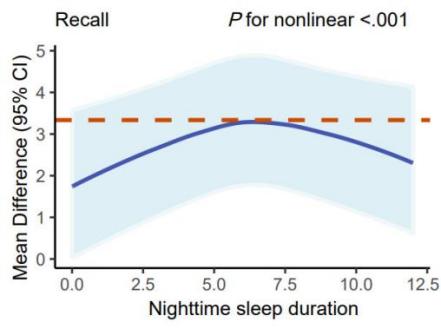


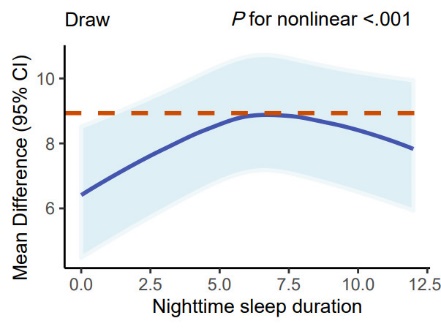

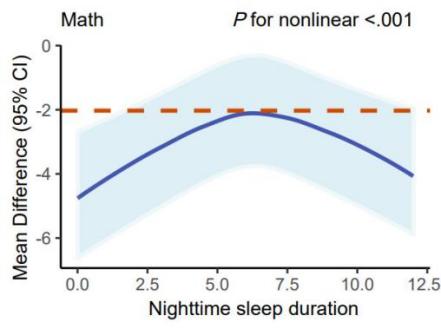


C Nap Duration


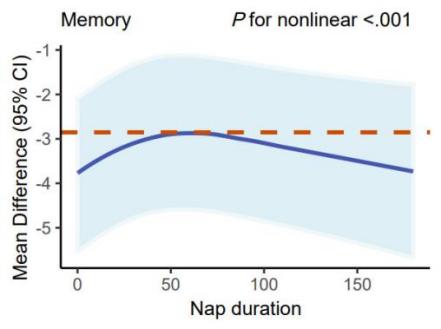

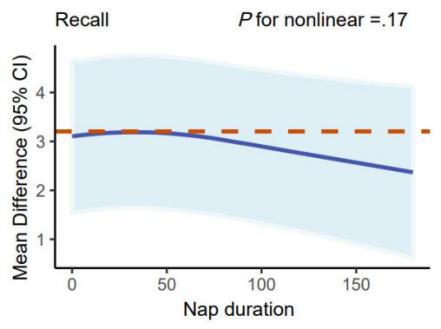


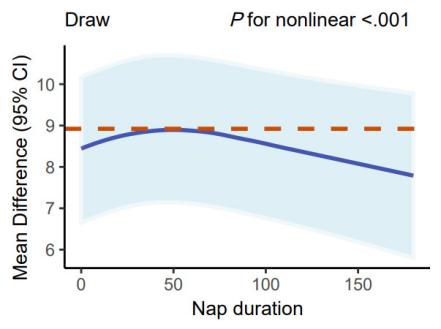

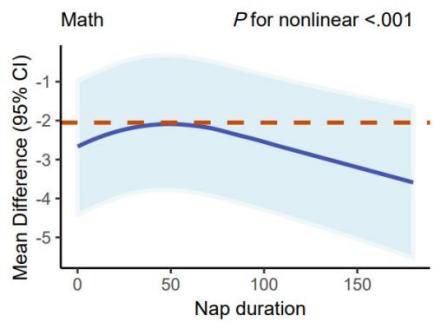


Figure S11: Restricted cubic spline models for the relationship between IC change and nap/total sleep duration ratio.


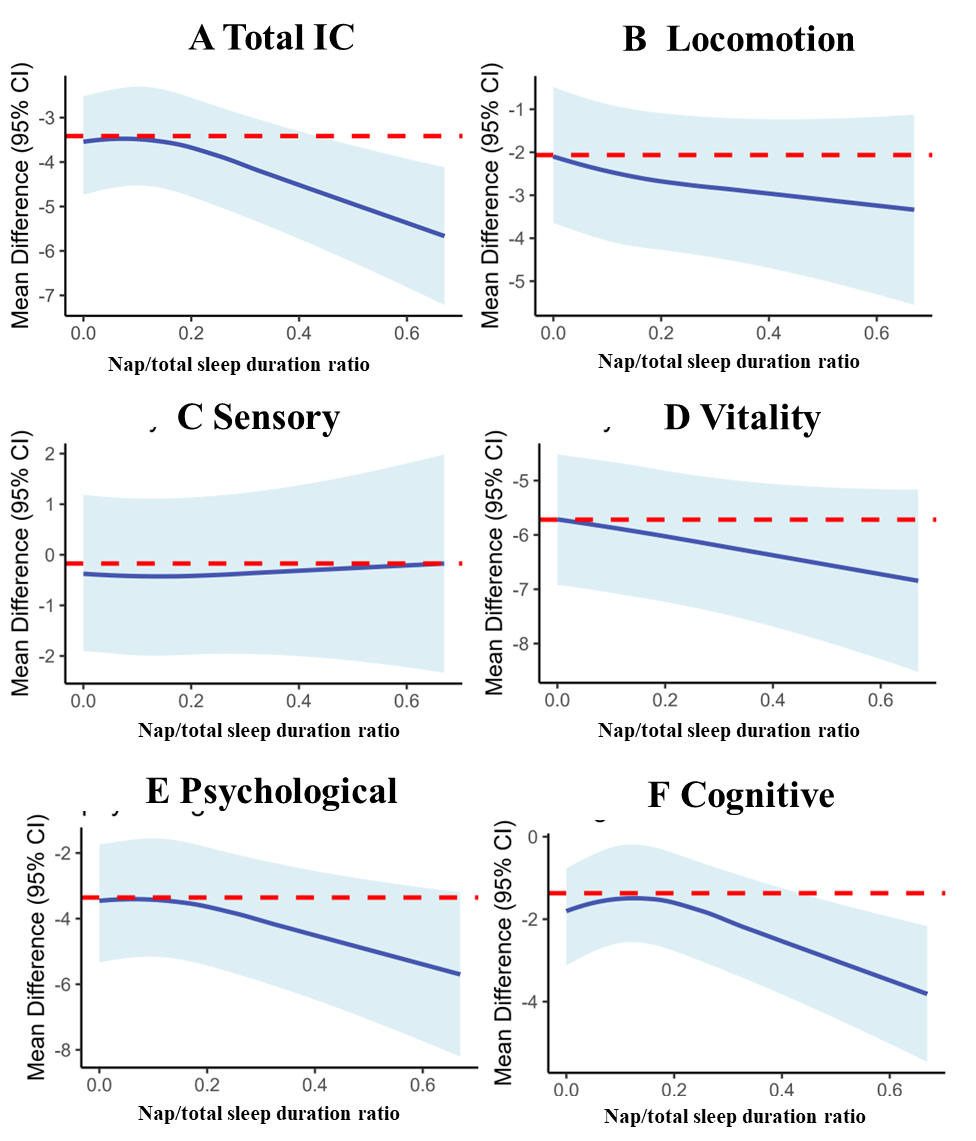


Figure S12. Subgroup analyses for the association between the total score of intrinsic capacity and less total sleep duration by gender, age, residence, BMI and median 2011 IC.


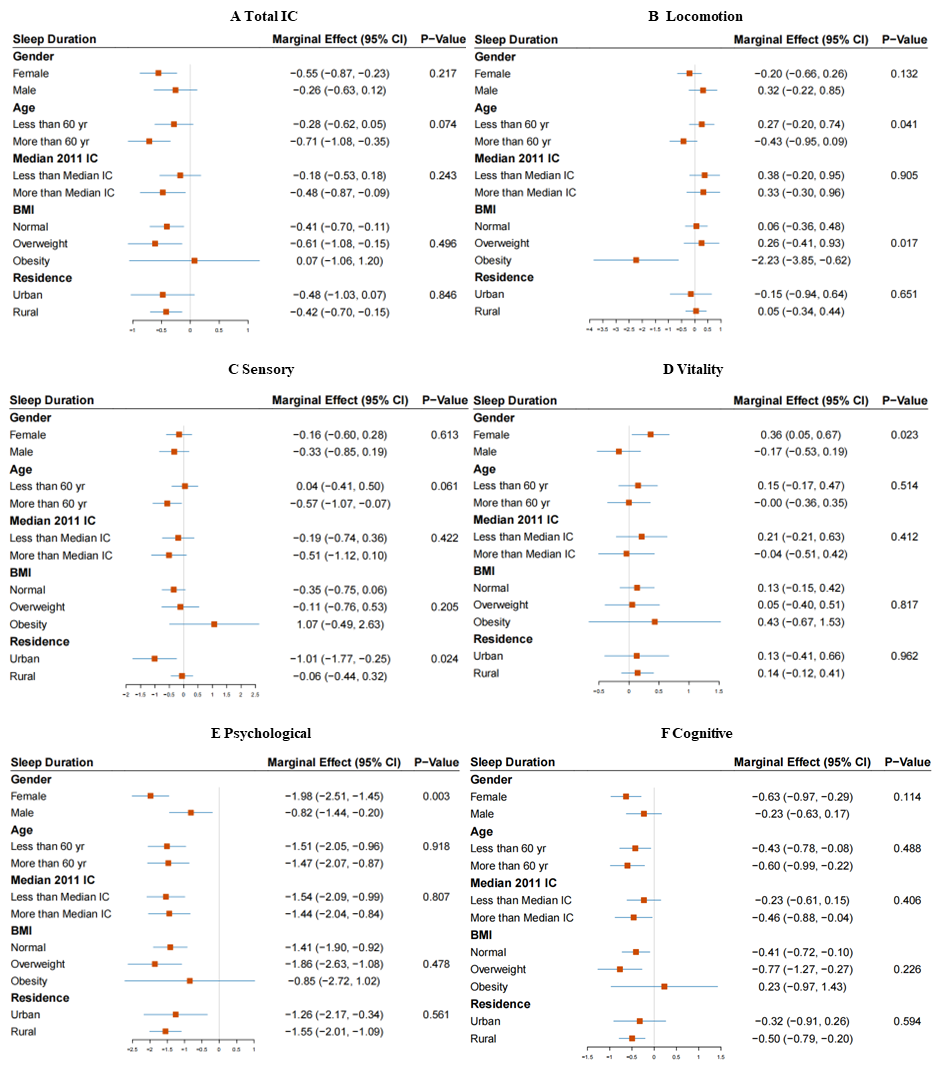


Figure S13. Subgroup analyses for the association between the total score of intrinsic capacity and over total sleep duration by gender, age, residence, BMI and median 2011 IC.


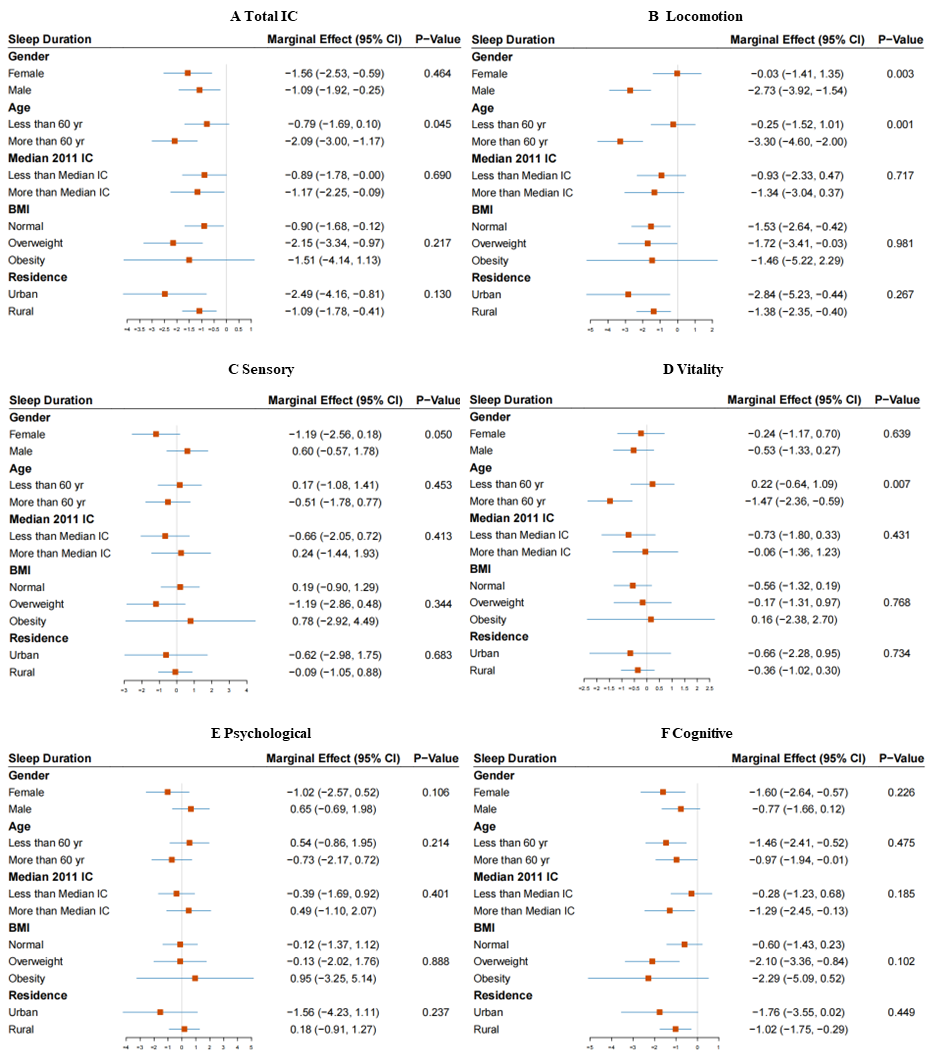


Figure S14. Subgroup analyses for the association between the total score of intrinsic capacity and less nighttime sleep duration by gender, age, residence, BMI and median 2011 IC.


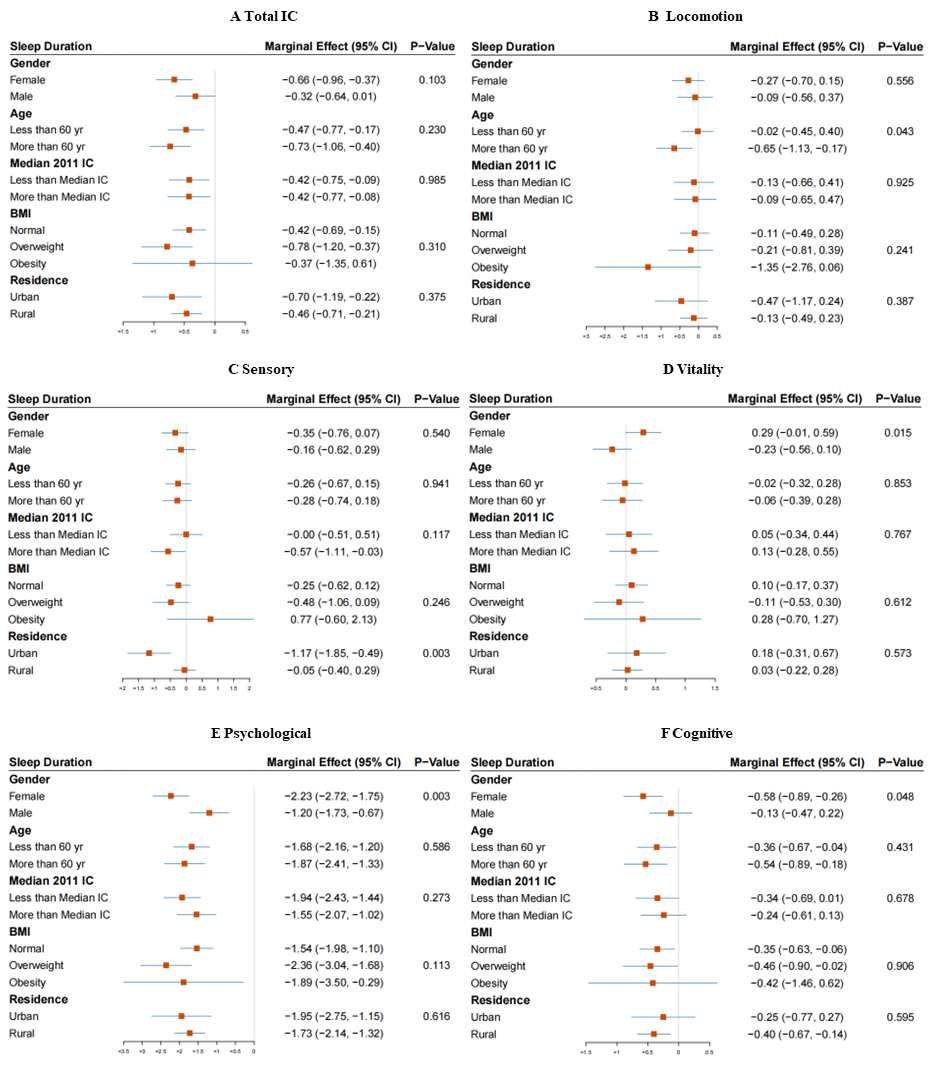


Figure S15. Subgroup analyses for the association between the total score of intrinsic capacity and over nighttime sleep duration by gender, age, residence, BMI and median 2011 IC.


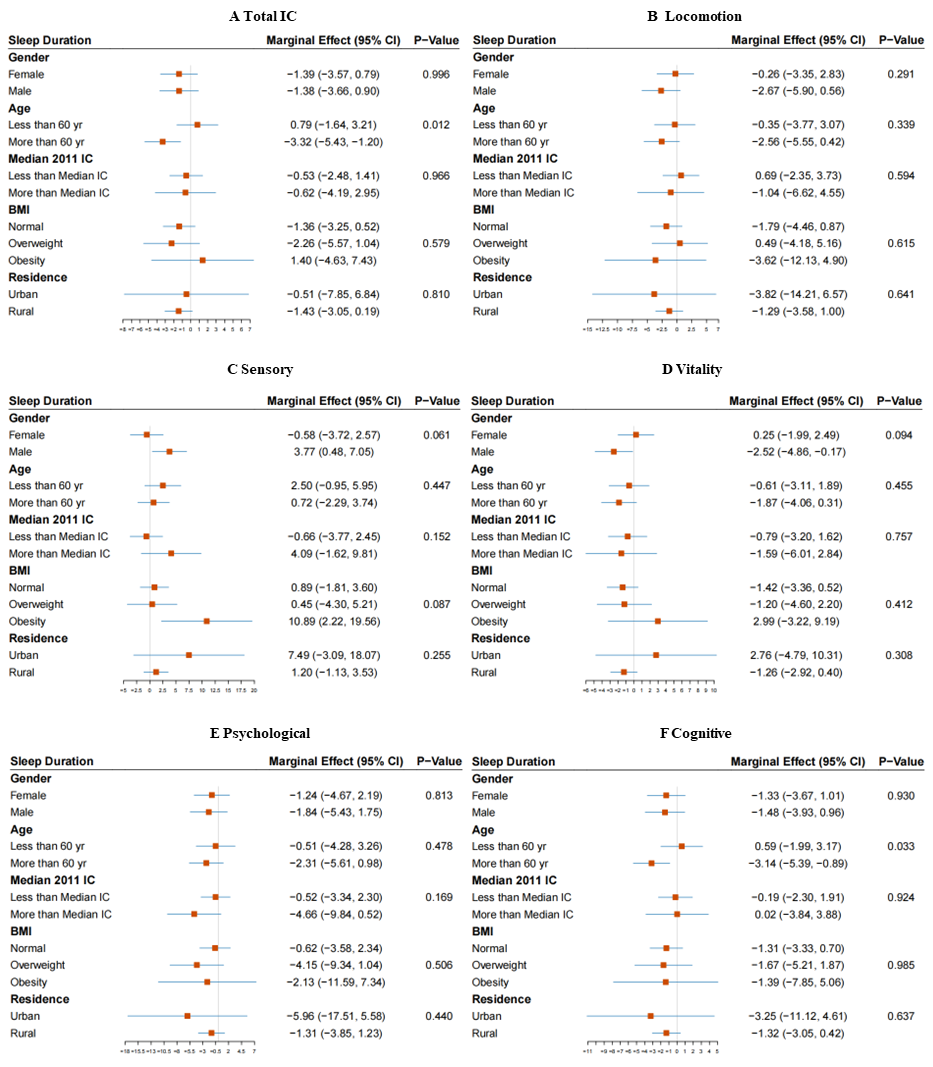


Figure S16. Subgroup analyses for the association between the total score of intrinsic capacity and moderate nap duration by gender, age, residence, BMI and median 2011 IC.


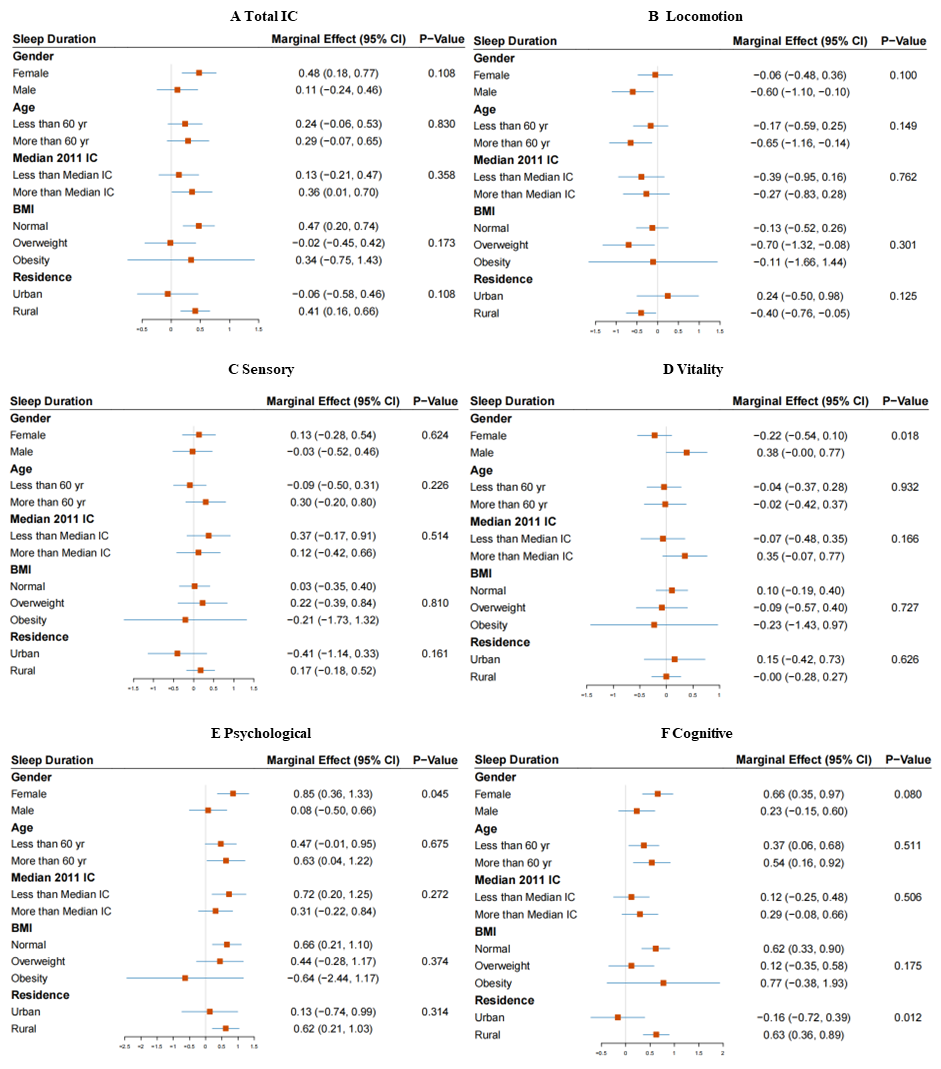


Figure S17. Subgroup analyses for the association between the total score of intrinsic capacity and over nap duration by gender, age, residence, BMI and median 2011 IC.


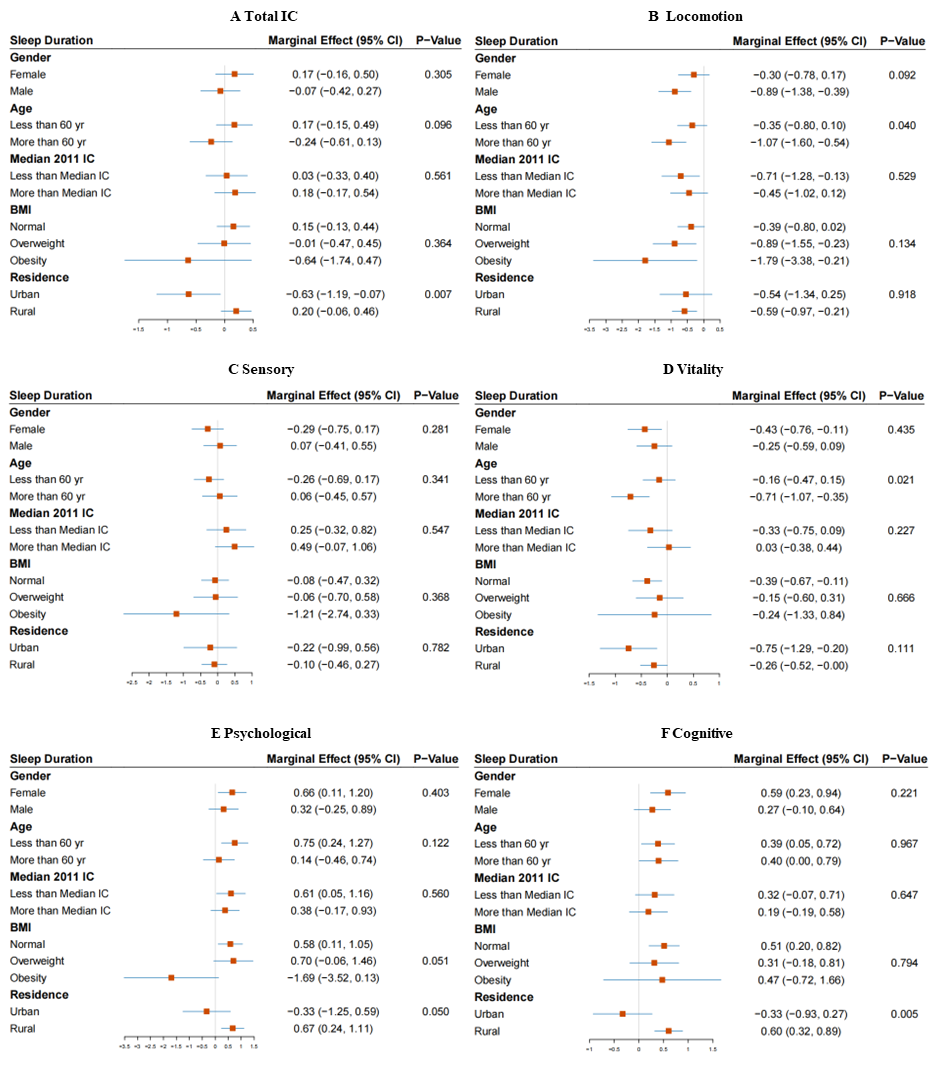


Figure S18. Subgroup analyses for the association between the intrinsic capacity and less/over nap duration by nighttime sleep duration.


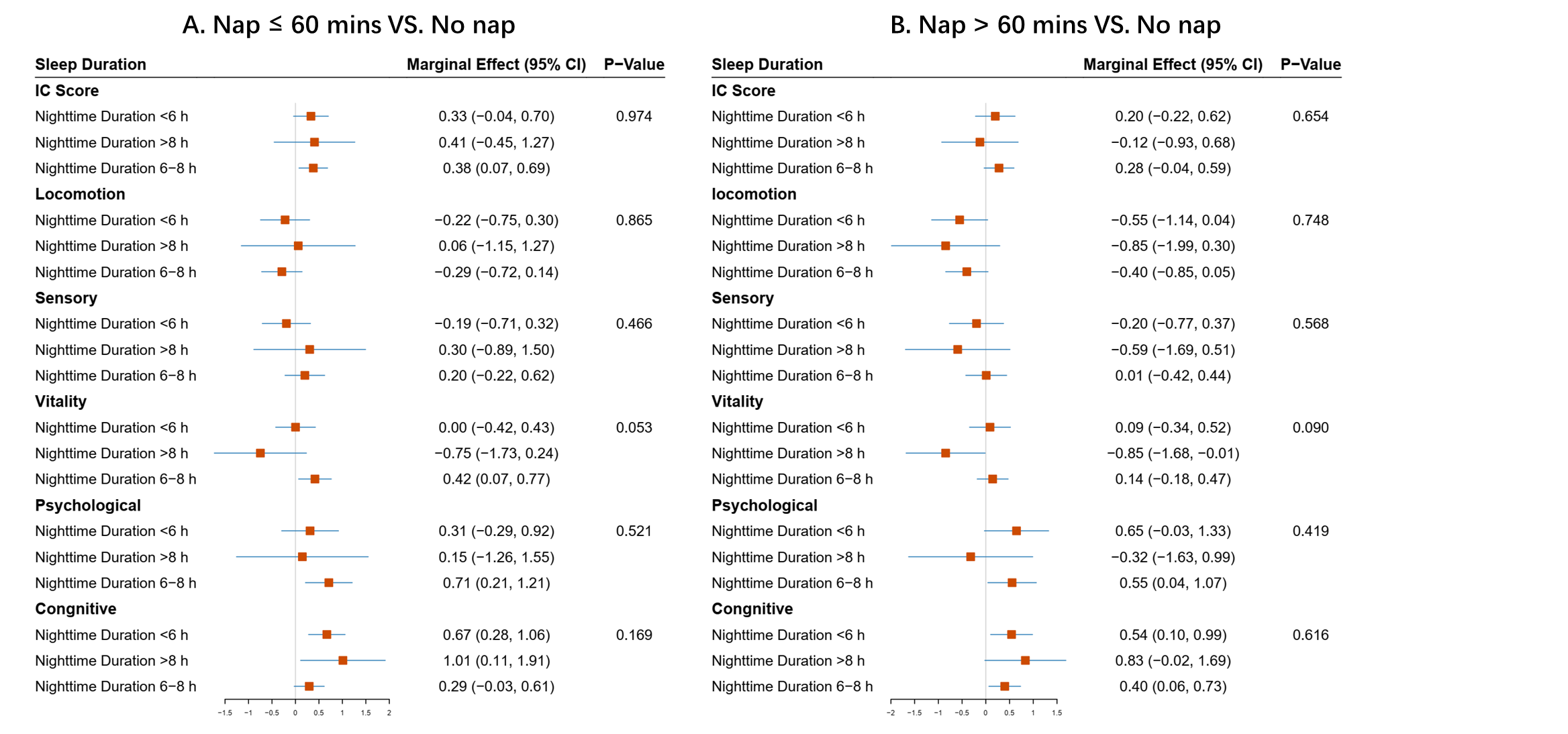


Table S1. Baseline sample characteristics according to sleep duration categories.

| Characteristics | Nighttime sleep duration, hour | | | | | Daytime napping, minute | | | |
| --- | --- | --- | --- | --- | --- | --- | --- | --- | --- |
|  | **<6** | **6 to ≤8** | **8-10** | **>10** | ***P* value** | **0** | **1 to ≤60** | **>60** | ***P* value** |
|  | **4410** | **6192** | **2068** | **156** |  | **4225** | **4640** | **3961** |  |
| Locomotion, mean (SD) |  |  |  |  |  |  |  |  |  |
| Gait | 0.23 (0.96) | -0.10 (0.83) | -0.13 (1.02) | -0.20 (1.09) | <0.001 | -0.22 (0.99) | -0.13 (0.88) | -0.10 (0.87) | <0.001 |
| Balance | -0.07 (1.00) | 0.08 (0.83) | 0.09 (0.83) | -0.07 (0.98) | <0.001 | 0.03 (0.90) | 0.03 (0.90) | 0.03 (0.89) | 0.923 |
| Chair | -0.13 (0.95) | 0.02 (0.85) | -0.01 (0.84) | -0.26 (0.99) | <0.001 | -0.05 (0.87) | -0.03 (0.90) | -0.04 (0.89) | 0.363 |
| Sensory |  |  |  |  |  |  |  |  |  |
| Hearing (%) |  |  |  |  |  |  |  |  |  |
| Good | 45 (1.0) | 84 (1.4) | 40 (1.9) | 2 (1.3) | <.001 | 1893 (44.8) | 2081 (44.8) | 1674 (42.3) | 0.033 |
| Fair | 1727 (39.2) | 2862 (46.2) | 995 (48.1) | 64 (41.0) |  | 62 (1.5) | 65 (1.4) | 44 (1.1) |  |
| Poor | 2638 (59.8) | 3246 (52.4) | 1033 (50.0) | 90 (57.7) |  | 2270 (53.7) | 2494 (53.8) | 2243 (56.6) |  |
| Vision Distance (%) |  |  |  |  |  |  |  |  |  |
| Good | 1393 (31.6) | 2467 (39.8) | 841 (40.7) | 62 (39.7) | <.001 | 1547 (36.6) | 1800 (38.8) | 1416 (35.7) | 0.046 |
| Fair | 47 (1.1) | 113 (1.8) | 47 (2.3) | 2 (1.3) |  | 74 (1.8) | 72 (1.6) | 63 (1.6) |  |
| Poor | 2970 (67.3) | 3612 (58.3) | 1180 (57.1) | 92 (59.0) |  | 2604 (61.6) | 2768 (59.7) | 2482 (62.7) |  |
| Vision Near (%) |  |  |  |  |  |  |  |  |  |
| Good | 1223 (27.7) | 2127 (34.4) | 768 (37.1) | 50 (32.1) | <.001 | 1423 (33.7) | 1506 (32.5) | 1239 (31.3) | 0.061 |
| Fair | 30 (0.7) | 80 (1.3) | 33 (1.6) | 2 (1.3) |  | 44 (1.0) | 63 (1.4) | 38 (1.0) |  |
| Poor | 3157 (71.6) | 3985 (64.4) | 1267 (61.3) | 104 (66.7) |  | 2758 (65.3) | 3071 (66.2) | 2684 (67.8) |  |
| Vitality, mean (SD) |  |  |  |  |  |  |  |  |  |
| Breath | -0.17 (0.96) | 0.07 (1.00) | -0.02 (1.00) | -0.26 (0.96) | <0.001 | -0.09 (0.99) | -0.03 (0.98) | 0.02 (1.01) | <0.001 |
| Grip | -0.05 (0.92) | 0.19 (1.05) | 0.16 (0.85) | 0.09 (0.90) | <0.001 | 0.00 (0.85) | 0.10 (1.00) | 0.21 (1.08) | <0.001 |
| Hemoglobin | 0.08 (1.04) | 0.19 (1.01) | 0.21 (1.09) | 0.33 (1.03) | <0.001 | 0.15 (1.10) | 0.12 (1.01) | 0.20 (0.99) | 0.001 |
| Psychological Capacity | -0.38 (1.03) | 0.20 (0.90) | 0.28 (0.83) | 0.04 (0.95) | <.001 | -0.09 (0.99) | -0.03 (0.98) | 0.02 (1.01) | <0.001 |
| Cognitive Capacity |  |  |  |  |  |  |  |  |  |
| Draw, complete (%) | -0.58 (1.00) | 0.25 (0.85) | 0.50 (0.73) | 0.53 (0.67) | <.001 | -0.06 (0.99) | 0.14 (0.91) | 0.11 (0.90) | <0.001 |
| Memory, mean (SD | 2529 (57.3) | 4232 (68.3) | 1296 (62.7) | 87 (55.8) | <.001 | -0.13 (1.05) | -0.04 (1.06) | -0.10 (1.05) | 0.001 |
| Recall, mean (SD) | -0.07 (0.98) | 0.18 (0.88) | 0.03 (0.96) | -0.24 (1.00) | <.001 | -0.22 (0.99) | -0.13 (0.88) | -0.10 (0.87) | <0.001 |
| Math, mean (SD) | -0.24 (1.03) | 0.02 (1.05) | -0.09 (1.07) | -0.22 (1.03) | <.001 | 0.03 (0.90) | 0.03 (0.90) | 0.03 (0.89) | 0.923 |
| Age, mean (SD), yr | 59.66 (9.27) | 57.37 (8.86) | 57.87 (9.59) | 59.47 (10.91) | <.001 | 57.76 (9.17) | 58.18 (9.00) | 58.90 (9.45) | <0.001 |
| Gender (% of male) | 1807 (41.0) | 3025 (48.9) | 1029 (49.8) | 83 (53.2) | <.001 | 1614 (38.2) | 2065 (44.5) | 2265 (57.2) | <0.001 |
| Residence (% of Urban) | 777 (17.6) | 1292 (20.9) | 364 (17.6) | 14 (9.0) | <0.001 | 688 (16.3) | 1002 (21.6) | 757 (19.1) | <0.001 |
| Education Level (%) |  |  |  |  |  |  |  |  |  |
| Primary school or lower | 3279 (74.4) | 3906 (63.1) | 1397 (67.6) | 124 (79.5) | <.001 | 3075 (72.8) | 3051 (65.8) | 2580 (65.1) | <.001 |
| Middle school | 771 (17.5) | 1452 (23.4) | 441 (21.3) | 24 (15.4) |  | 766 (18.1) | 1000 (21.6) | 922 (23.3) |  |
| High school or higher | 360 (8.2) | 834 (13.5) | 230 (11.1) | 8 (5.1) |  | 384 (9.1) | 589 (12.7) | 459 (11.6) |  |
| Marry status (%) | 4364 (99.0) | 6156 (99.4) | 2051 (99.2) | 152 (97.4) | 0.004 | 4183 (99.0) | 4613 (99.4) | 3927 (99.1) | .015 |
| ^c^Income, mean (SD), CNY | 6399.35  (7836.65) | 7193.76  (8369. 32) | 7187.34  (8824. 48) | 7029.81  (8941.60) | <.001 | 6732.42  (7981.26) | 6979.42  (8434. 27) | 7042.67  (8413. 01) | 0.195 |
| Smoking (% of Yes) | 1573 (35.7) | 2489 (40.2) | 797 (38.5) | 65 (41.7) | <0.001 | 1433 (33.9) | 1637 (35.3) | 1854 (46.8) | <0.001 |
| Drinking (% of Yes) | 1344 (30.5) | 2114 (34.1) | 695 (33.6) | 47 (30.1) | 0.001 | 1160 (27.5) | 1498 (32.3) | 1542 (38.9) | <0.001 |
| ^d^Physical Activities, MET |  |  |  |  |  |  |  |  |  |
| Vigorous activities (%) | 1469 (33.3) | 2271 (36.7) | 748 (36.2) | 62 (39.7) | 0.002 | 1566 (37.1) | 1619 (34.9) | 1365 (34.5) | 0.028 |
| Middle activities (%) | 2694 (61.1) | 3861 (62.4) | 1289 (62.3) | 91 (58.3) | 0.428 | 2686 (63.6) | 2872 (61.9) | 2377 (60.0) | 0.004 |
| Leisure activities (%) | 3824 (86.7) | 5336 (86.2) | 1768 (85.5) | 123 (78.8) | 0.031 | 3613 (85.5) | 4016 (86.6) | 3422 (86.4) | 0.324 |
| Tranquilizers or sleeping pills (%) | 57 (1.3) | 28 (0.5) | 11 (0.5) | 2 (1.3) | <0.001 | 29 (0.7) | 43 (0.9) | 26 (0.7) | 0.278 |
| Sleep quality (%) |  |  |  |  |  |  |  |  |  |
| Good | 1043 (23.7) | 3557 (57.4) | 1535 (74.2) | 117 (75.0) | <.001 | 1841 (43.6) | 2207 (47.6) | 2204 (55.6) | <.001 |
| Fair | 1640 (37.2) | 2005 (32.4) | 396 (19.1) | 33 (21.2) |  | 1355 (32.1) | 1548 (33.4) | 1171 (29.6) |  |
| Poor | 1727 (39.2) | 630 (10.2) | 137 (6.6) | 6 (3.8) |  | 1029 (24.4) | 885 (19.1) | 586 (14.8) |  |
| Mental health (mean (SD)) | -0.38 (1.03) | 0.20 (0.90) | 0.28 (0.83) | 0.04 (0.95) | <0.001 | 20.69 (6.74) | 21.57 (6.34) | 21.91 (6.21) | <0.001 |
| Memory |  |  |  |  |  |  |  |  |  |
| Good | 594 (13.5) | 1259 (20.3) | 373 (18.0) | 16 (10.3) | <.001 | 680 (16.1) | 876 (18.9) | 686 (17.3) | <.001 |
| Fair | 2376 (53.9) | 3498 (56.5) | 1128 (54.5) | 88 (56.4) |  | 2290 (54.2) | 2565 (55.3) | 2235 (56.4) |  |
| Poor | 1440 (32.7) | 1435 (23.2) | 567 (27.4) | 52 (33.3) |  | 1255 (29.7) | 1199 (25.8) | 1040 (26.3) |  |
| Disability (%) | 187 (4.2) | 220 (3.6) | 87 (4.2) | 7 (4.5) | 0.261 | 163 (3.9) | 172 (3.7) | 166 (4.2) | 0.504 |
| ^b^BMI mean (SD), kg/m^2^ | 23.28 (3.59) | 23.70 (3.58) | 23.64 (3.47) | 23.13 (3.49) | <0.001 | 23.20 (3.51) | 23.72 (3.59) | 23.70 (3.59) | <0.001 |
| Chronic Disease (%) |  |  |  |  |  |  |  |  |  |
| Hypertension | 1110 (25.2) | 1368 (22.1) | 482 (23.3) | 33 (21.2) | 0.003 | 850 (20.1) | 1136 (24.5) | 1007 (25.4) | <0.001 |
| Dyslipidemia | 447 (10.1) | 541 (8.7) | 180 (8.7) | 13 (8.3) | 0.073 | 287 (6.8) | 478 (10.3) | 416 (10.5) | <0.001 |
| Diabetes | 260 (5.9) | 334 (5.4) | 91 (4.4) | 8 (5.1) | 0.103 | 159 (3.8) | 301 (6.5) | 233 (5.9) | <0.001 |
| Cancer | 56 (1.3) | 52 (0.8) | 16 (0.8) | 0 (0.0) | 0.055 | 52 (1.2) | 43 (0.9) | 29 (0.7) | 0.066 |
| Chronic lung diseases | 550 (12.5) | 518 (8.4) | 178 (8.6) | 13 (8.3) | <0.001 | 409 (9.7) | 448 (9.7) | 402 (10.1) | 0.698 |
| Liver diseases | 221 (5.0) | 215 (3.5) | 62 (3.0) | 6 (3.8) | <0.001 | 164 (3.9) | 209 (4.5) | 131 (3.3) | 0.017 |
| Heart diseases | 649 (14.7) | 649 (10.5) | 181 (8.8) | 18 (11.5) | <0.001 | 453 (10.7) | 598 (12.9) | 446 (11.3) | 0.004 |
| Stroke | 99 (2.2) | 96 (1.6) | 39 (1.9) | 2 (1.3) | 0.067 | 72 (1.7) | 83 (1.8) | 81 (2.0) | 0.492 |
| Kidney diseases | 372 (8.4) | 312 (5.0) | 102 (4.9) | 11 (7.1) | <0.001 | 275 (6.5) | 287 (6.2) | 235 (5.9) | 0.556 |
| Digestive diseases | 1318 (29.9) | 1263 (20.4) | 383 (18.5) | 34 (21.8) | <0.001 | 1056 (25.0) | 1081 (23.3) | 861 (21.7) | 0.002 |
| Psychiatric problems | 72 (1.6) | 51 (0.8) | 13 (0.6) | 4 (2.6) | <.001 | 43 (1.0) | 57 (1.2) | 40 (1.0) | 0.532 |
| Memory-related diseases | 60 (1.4) | 53 (0.9) | 23 (1.1) | 4 (2.6) | 0.026 | 41 (1.0) | 52 (1.1) | 47 (1.2) | 0.624 |
| Arthritis or rheumatism | 1935 (43.9) | 1851 (29.9) | 594 (28.7) | 67 (42.9) | <0.001 | 1568 (37.1) | 1614 (34.8) | 1265 (31.9) | <0.001 |
| Asthma | 189 (4.3) | 166 (2.7) | 65 (3.1) | 8 (5.1) | <0.001 | 140 (3.3) | 139 (3.0) | 149 (3.8) | 0.142 |

Note: Inconsistencies arise in some values due to rounding.

a: The interviewer recorded respondent’s gender as either female or male.

b: BMI, body mass index.

c: Chinese yuan.

d: Physical activity was defined according to each cohort’s questionnaire. Vigorous activities make you breathe much harder than normal and may include heavy lifting, digging, plowing, aerobics, fast bicycling, and cycling with a heavy load in a usual week; Moderate physical activities make you breathe somewhat harder than normal and may include carrying light loads, bicycling at a regular pace, or mopping the floor in a usual week; leisure activities includes at work and at home, walking to travel from place to place, and any other walking that you might do solely for recreation, sport, exercise.

Table S2. Sensitivity analysis: Model 1 was used to evaluate the original IC total score of the middle-aged and elderly population's sleep duration in 2011.

| Intrinsic capacity (IC) and five subdomains | Nighttime sleep duration, hour | | | | |  | Daytime napping, minute | | | | |  | Total sleep duration, hour | | | | |
| --- | --- | --- | --- | --- | --- | --- | --- | --- | --- | --- | --- | --- | --- | --- | --- | --- | --- |
|  | <6 | |  | >10 | |  | 1 to ≤60 | |  | >60 | |  | <6 | |  | >10 | |
|  | Marginal effect  (95%CI) | *P*  value |  | Marginal effect (95%CI) | *P*  value |  | Marginal effect (95%CI) | *P*  value |  | Marginal effect (95%CI) | *P* value |  | Marginal effect (95%CI) | *P* value |  | Marginal effect (95%CI) | *P* value |
| IC total score | -0.51  (-0.74, -0.28) | <.001 |  | -1.43  (-2.27, -0.58) | <.001 |  | 0.32  (0.10, 0.55) | <.001 |  | 0.05  (-0.19, 0.29) | 0.66 |  | -0.43  (-0.68, -0.18) | <.001 |  | -1.12  (-1.61, -0.64) | <.001 |
| Locomotion | -0.19  (-0.52, 0.14) | 0.25 |  | -1.93  (-3.13, -0.73) | <.001 |  | -0.28  (-0.61, 0.04) | .08 |  | -0.58  (-0.93, -0.24) | <.001 |  | 0.01  (-0.35, 0.38) | 0.94 |  | -1.22  (-1.92, -0.52) | <.001 |
| Sensory | -0.26  (-0.58, 0.06) | 0.11 |  | 0.53  (-0.69, 1.75) | .39 |  | 0.07  (-0.25, 0.38) | .68 |  | -0.12  (-0.45, 0.22) | 0.49 |  | -0.23  (-0.58, 0.12) | 0.20 |  | -0.46  (-1.14, 0.23) | .19 |
| Vitality | 0.06  (-0.17, 0.29) | 0.63 |  | -0.23  (-1.10, 0.63) | .60 |  | 0.03  (-0.22, 0.27) | .84 |  | -0.34  (-0.58, -0.11) | <.001 |  | 0.14  (-0.11, 0.39) | 0.26 |  | -0.38  (-0.85, 0.09) | .11 |
| Psychological | -1.77  (-2.15, -1.39) | <.001 |  | -0.80  (-2.12, 0.53) | 0.24 |  | 0.53  (0.15, 0.90) | .01 |  | 0.50  (0.10, 0.89) | 0.01 |  | -1.50  (-1.92, -1.08) | <.001 |  | 0.34  (-0.43 1.12) | .38 |
| Cognitive | -0.37  (-0.62, -0.13) | <.001 |  | -1.70  (-2.61,0.79) | <.001 |  | 0.48  (0.24, 0.72) | <.001 |  | 0.44  (0.18, 0.70) | <.001 |  | -0.47  (-0.73, -0.20) | <.001 |  | -1.10  (-1.62, -0.58) | <.001 |

Multivariate linear regression analysis adjusted 29 covariates including demographic variables (age, gender, residence, education level, and married status )+ socioeconomic status (annual household income) +lifestyle (drinking history, smoking history, physical activity level, memory, mental health, sleep quality ,and tranquilizers or sleeping drugs) +health status (BMI, physical disabilities, hypertension, dyslipidemia, diabetes, cancer, chronic lung diseases, liver disease, heart diseases, stroke, kidney diseases, digestive diseases, psychiatric problems, memory-related diseases, arthritis or rheumatism, and asthma). The IC total score was adjusted using the residual method.

Table S3. Sensitivity analysis: model 2 was used to evaluate the relationship between IC and sleep duration in the elderly population after excluding the use of sedatives or sleeping pills.

| Intrinsic capacity (IC) and five subdomains | Nighttime sleep duration, hour | | | | |  | Daytime napping, minute | | | | |  | Total sleep duration, hour | | | | |
| --- | --- | --- | --- | --- | --- | --- | --- | --- | --- | --- | --- | --- | --- | --- | --- | --- | --- |
|  | <6 | |  | >10 | |  | 1 to ≤60 | |  | >60 | |  | <6 | |  | >10 | |
|  | Marginal effect  (95%CI) | *P*  value |  | Marginal effect (95%CI) | *P*  value |  | Marginal effect (95%CI) | *P*  value |  | Marginal effect (95%CI) | *P* value |  | Marginal effect (95%CI) | *P* value |  | Marginal effect (95%CI) | *P* value |
| IC total score | -0.51  (-0.74, -0.28) | <.001 |  | -1.31  (-2.16, -0.46) | <.001 |  | 0.34  (0.11, 0.56) | <.001 |  | 0.07  (-0.17, 0.31) | .57 |  | -0.42  (-0.67, -0.17) | <.001 |  | -1.04  (-1.53, -0.56) | <.001 |
| Locomotion | -0.20  (-0.53, 0.13) | .23 |  | -1.88  (-3.08, -0.68) | <.001 |  | -0.25  (-0.57, 0.07) | .13 |  | -0.55  (-0.89, -0.21) | <.001 |  | 0.01  (-0.35, 0.37) | .95 |  | -1.16  (-1.85, -0.46) | <.001 |
| Sensory | -0.26  (-0.58, 0.06) | .11 |  | 0.65  (-0.58, 1.88) | .30 |  | 0.06  (-0.26, 0.38) | .70 |  | -0.13  (-0.47, 0.20) | .43 |  | -0.22  (-0.57, 0.13) | .22 |  | -0.41  (-1.10, 0.28) | .25 |
| Vitality | 0.05  (-0.18, 0.28) | .69 |  | -0.21  (-1.09, 0.66) | .63 |  | 0.03  (-0.22, 0.28) | .81 |  | -0.34  (-0.57, -0.10) | .01 |  | 0.12  (-0.13, 0.37) | .34 |  | -0.35  (-0.82, 0.13) | .15 |
| Psychological | -1.73  (-2.11, -1.36) | <.001 |  | -0.57  (-1.91, 0.76) | .40 |  | 0.54  (0.17, 0.92) | <.001 |  | 0.51  (0.12, 0.91) | .01 |  | -1.45  (-1.87, -1.02) | <.001 |  | 0.47  (-0.31, 1.25) | .23 |
| Cognitive | -0.38  (-0.62, -0.13) | <.001 |  | -1.66  (-2.57, -0.74) | <.001 |  | 0.48  (0.24, 0.72) | <.001 |  | 0.45  (0.19, 0.71) | <.001 |  | -0.45  (-0.72, -0.18) | <.001 |  | -1.05  (-1.57, -0.52) | <.001 |

Multivariate linear regression analysis adjusted 29 covariates including demographic variables (age, gender, residence, education level, and married status )+ socioeconomic status (annual household income) +lifestyle (drinking history, smoking history, physical activity level, memory, mental health, sleep quality ,and tranquilizers or sleeping drugs) +health status (BMI, physical disabilities, hypertension, dyslipidemia, diabetes, cancer, chronic lung diseases, liver disease, heart diseases, stroke, kidney diseases, digestive diseases, psychiatric problems, memory-related diseases, arthritis or rheumatism, and asthma). The IC total score was adjusted using the residual method.

Table S4. Sensitivity analysis: model 3 was used to evaluate the relationship between IC and sleep duration in the elderly population after excluding the respondents with physical disabilities.

| Intrinsic capacity (IC) and five subdomains | Nighttime sleep duration, hour | | | | |  | Daytime napping, minute | | | | |  | Total sleep duration, hour | | | | |
| --- | --- | --- | --- | --- | --- | --- | --- | --- | --- | --- | --- | --- | --- | --- | --- | --- | --- |
|  | <6 | |  | >10 | |  | 1 to ≤60 | |  | >60 | |  | <6 | |  | >10 | |
|  | Marginal effect  (95%CI) | *P*  value |  | Marginal effect (95%CI) | *P*  value |  | Marginal effect (95%CI) | *P*  value |  | Marginal effect (95%CI) | *P* value |  | Marginal effect (95%CI) | *P* value |  | Marginal effect (95%CI) | *P* value |
| IC total score | -0.60  (-0.83, -0.36) | <.001 |  | -1.41  (-2.28, -0.54) | <.001 |  | 0.29  (0.06, 0.52) | .01 |  | 0.05  (-0.20, 0.29) | .71 |  | -0.50  (-0.75, -0.24) | <.001 |  | -1.18  (-1.68, -0.68) | <.001 |
| Locomotion | -0.25  (-0.58, 0.08) | .13 |  | -1.83  (-3.03, -0.63) | <.001 |  | -0.34  (-0.67, -0.02) | .04 |  | -0.64  (-0.99, -0.30) | <.001 |  | -0.05  (-0.41, 0.31) | .79 |  | -1.32  (-2.03, -0.62) | <.001 |
| Sensory | -0.30  (-0.63, 0.02) | .07 |  | 0.27  (-0.98, 1.52) | 0.67 |  | 0.01  (-0.31, 0.34) | .94 |  | -0.21  (-0.55, 0.13) | .23 |  | -0.28  (-0.64, 0.08) | .12 |  | -0.56  (-1.26, 0.14) | .12 |
| Vitality | 0.01  (-0.22, 0.25) | .93 |  | -0.18  (-1.07, 0.71) | .70 |  | -0.01  (-0.27, 0.24) | .93 |  | -0.34  (-0.58, -0.10) | .01 |  | 0.09  (-0.16, 0.34) | .48 |  | -0.41  (-0.89, 0.07) | .09 |
| Psychological | -1.79  (-2.17, -1.41) | <.001 |  | -0.74  (-2.10, 0.61) | .28 |  | 0.54  (0.16, 0.92) | .01 |  | 0.47  (0.07, 0.88) | .02 |  | -1.51  (-1.94, -1.08) | <.001 |  | 0.23  (-0.56, 1.02) | .57 |
| Cognitive | -0.43  (-0.68, -0.19) | <.001 |  | -1.65  (-2.58, -0.73) | <.001 |  | 0.50  (0.25, 0.74) | <.001 |  | 0.47  (0.20, 0.73) | <.001 |  | -0.50  (-0.78, -0.23) | <.001 |  | -1.10  (-1.63, -0.57) | <.001 |

Multivariate linear regression analysis adjusted 29 covariates including demographic variables (age, gender, residence, education level, and married status )+ socioeconomic status (annual household income) +lifestyle (drinking history, smoking history, physical activity level, memory, mental health, sleep quality ,and tranquilizers or sleeping drugs) +health status (BMI, physical disabilities, hypertension, dyslipidemia, diabetes, cancer, chronic lung diseases, liver disease, heart diseases, stroke, kidney diseases, digestive diseases, psychiatric problems, memory-related diseases, arthritis or rheumatism, and asthma). The IC total score was adjusted using the residual method.

Table S5. Sensitivity analysis: model 4 was used to evaluate the relationship between IC and sleep duration in the elderly population after excluding the total sleep duration value of zero.

| Intrinsic capacity (IC) and five subdomains | Nighttime sleep duration, hour | | | | |  | Daytime napping, minute | | | | |  | Total sleep duration, hour | | | | |
| --- | --- | --- | --- | --- | --- | --- | --- | --- | --- | --- | --- | --- | --- | --- | --- | --- | --- |
|  | <6 | |  | >10 | |  | 1 to ≤60 | |  | >60 | |  | <6 | |  | >10 | |
|  | Marginal effect  (95%CI) | *P*  value |  | Marginal effect (95%CI) | *P*  value |  | Marginal effect (95%CI) | *P*  value |  | Marginal effect (95%CI) | *P* value |  | Marginal effect (95%CI) | *P* value |  | Marginal effect (95%CI) | *P* value |
| IC total score | -0.51  (-0.74, -0.28) | <.001 |  | -1.38  (-2.22, -0.54) | <.001 |  | 0.30  (0.07, 0.52) | .01 |  | 0.05  (-0.19, 0.29) | .68 |  | -0.43  (-0.68, -0.18) | <.001 |  | -1.10  (-1.59, -0.62) | <.001 |
| Locomotion | -0.20  (-0.53, 0.13) | .23 |  | -1.93  (-3.13, -0.73) | <.001 |  | -0.30  (-0.63, 0.02) | .06 |  | -0.58  (-0.93, -0.24) | <.001 |  | 0.05  (-0.31, 0.41) | .80 |  | -1.21  (-1.90, -0.52) | <.001 |
| Sensory | -0.26  (-0.58, 0.06) | .11 |  | 0.53  (-0.69, 1.75) | .39 |  | 0.07  (-0.25, 0.38) | .69 |  | -0.12  (-0.45, 0.21) | .48 |  | -0.23  (-0.58, 0.13) | .21 |  | -0.46  (-1.14, 0.23) | .19 |
| Vitality | 0.05  (-0.15, 0.25) | .62 |  | -0.09  (-0.81, 0.64) | .82 |  | -0.06  (-0.26, 0.14) | .54 |  | -0.35  (-0.57, -0.13) | <.001 |  | 0.10  (-0.12, 0.31) | .38 |  | -0.34  (-0.76, 0.07) | .11 |
| Psychological | -1.77  (-2.14, -1.39) | <.001 |  | -0.80  (-2.13, 0.53) | .24 |  | 0.52  (0.15, 0.90) | .01 |  | 0.50  (0.10, 0.89) | .01 |  | -1.49  (-1.91, -1.07) | <.001 |  | 0.34  (-0.43, 1.12) | .38 |
| Cognitive | -0.38  (-0.62, -0.13) | <.001 |  | -1.70  (-2.61, -0.79) | <.001 |  | 0.48  (0.24, 0.72) | <.001 |  | 0.44  (0.18, 0.70) | <.001 |  | -0.47  (-0.74, -0.20) | <.001 |  | -1.10  (-1.62, -0.58) | <.001 |

Multivariate linear regression analysis adjusted 29 covariates including demographic variables (age, gender, residence, education level, and married status )+ socioeconomic status (annual household income) +lifestyle (drinking history, smoking history, physical activity level, memory, mental health, sleep quality ,and tranquilizers or sleeping drugs) +health status (BMI, physical disabilities, hypertension, dyslipidemia, diabetes, cancer, chronic lung diseases, liver disease, heart diseases, stroke, kidney diseases, digestive diseases, psychiatric problems, memory-related diseases, arthritis or rheumatism, and asthma). The IC total score was adjusted using the residual method.

Table S6. Sensitivity analysis: model 5 was used to evaluate the relationship between IC and sleep duration in the elderly population after excluding the extreme IC total score.

| Intrinsic capacity (IC) and five subdomains | Nighttime sleep duration, hour | | | | |  | Daytime napping, minute | | | | |  | Total sleep duration, hour | | | | |
| --- | --- | --- | --- | --- | --- | --- | --- | --- | --- | --- | --- | --- | --- | --- | --- | --- | --- |
|  | <6 | |  | >10 | |  | 1 to ≤60 | |  | >60 | |  | <6 | |  | >10 | |
|  | Marginal effect  (95%CI) | *P*  value |  | Marginal effect (95%CI) | *P*  value |  | Marginal effect (95%CI) | *P*  value |  | Marginal effect (95%CI) | *P* value |  | Marginal effect (95%CI) | *P* value |  | Marginal effect (95%CI) | *P* value |
| IC total score | -0.51  (-0.74, -0.28) | <.001 |  | -1.43  (-2.27, -0.58) | <.001 |  | 0.32  (0.10, 0.55) | <.001 |  | 0.05  (-0.19, 0.29) | .66 |  | -0.43  (-0.68, -0.18) | <.001 |  | -1.12  (-1.61, -0.64) | <.001 |
| Locomotion | -0.18  (-0.51, 0.15) | .28 |  | -1.93  (-3.13, -0.73) | <.001 |  | -0.30  (-0.62, 0.02) | .07 |  | -0.60  (-0.94, -0.25) | <.001 |  | 0.03  (-0.33, 0.39) | .88 |  | -1.22  (-1.92, -0.52) | <.001 |
| Sensory | -0.27  (-0.59, 0.05) | .10 |  | 0.53  (-0.69, 1.75) | .39 |  | 0.07  (-0.25, 0.39) | .66 |  | -0.11  (-0.45, 0.22) | .50 |  | -0.24  (-0.59, 0.11) | .19 |  | -0.46  (-1.14, 0.23) | .19 |
| Vitality | 0.06  (-0.17, 0.29) | .63 |  | -0.23  (-1.10, 0.63) | .60 |  | 0.03  (-0.22, 0.27) | .83 |  | -0.34  (-0.58, -0.11) | <.001 |  | 0.14  (-0.11, 0.39) | .26 |  | -0.38  (-0.85, 0.09) | .11 |
| Psychological | -1.78  (-2.16, -1.41) | <.001 |  | -0.80  (-2.12, 0.53) | .24 |  | 0.55  (0.17, 0.92) | <.001 |  | 0.51  (0.12, 0.91) | .01 |  | -1.52  (-1.94, -1.10) | <.001 |  | 0.34  (-0.43, 1.12) | .38 |
| Cognitive | -0.37  (-0.62, -0.13) | <.001 |  | -1.70  (-2.61, -0.79) | <.001 |  | 0.48  (0.24, 0.72) | <.001 |  | 0.43  (0.18, 0.69) | <.001 |  | -0.47  (-0.74, -0.20) | <.001 |  | -1.10  (-1.62, -0.58) | <.001 |

Multivariate linear regression analysis adjusted 29 covariates including demographic variables (age, gender, residence, education level, and married status )+ socioeconomic status (annual household income) +lifestyle (drinking history, smoking history, physical activity level, memory, mental health, sleep quality ,and tranquilizers or sleeping drugs) +health status (BMI, physical disabilities, hypertension, dyslipidemia, diabetes, cancer, chronic lung diseases, liver disease, heart diseases, stroke, kidney diseases, digestive diseases, psychiatric problems, memory-related diseases, arthritis or rheumatism, and asthma). The IC total score was adjusted using the residual method.

Table S7. Sensitivity analysis: model 5 was used to evaluate the relationship between IC and sleep duration in the elderly population who completed receding position procedures.

| Intrinsic capacity (IC) and five subdomains | Nighttime sleep duration, hour | | | | |  | Daytime napping, minute | | | | |  | Total sleep duration, hour | | | | |
| --- | --- | --- | --- | --- | --- | --- | --- | --- | --- | --- | --- | --- | --- | --- | --- | --- | --- |
|  | <6 | |  | >10 | |  | 1 to ≤60 | |  | >60 | |  | <6 | |  | >10 | |
|  | Marginal effect  (95%CI) | *P*  value |  | Marginal effect (95%CI) | *P*  value |  | Marginal effect (95%CI) | *P*  value |  | Marginal effect (95%CI) | *P* value |  | Marginal effect (95%CI) | *P* value |  | Marginal effect (95%CI) | *P* value |
| IC total score | -0.43  (-0.68, -0.19) | <.001 |  | -1.32  (-2.24, -0.39) | .01 |  | 0.31  (0.06, 0.55) | <.01 |  | 0.06  (-0.20, 0.32) | .67 |  | -0.42  (-0.69, -0.15) | <.001 |  | -1.09  (-1.62, -0.56) | <.001 |
| Locomotion | -0.12  (-0.48, 0.23) | .50 |  | -1.66  (-2.99, -0.33) | .01 |  | -0.27  (-0.62, 0.08) | .14 |  | -0.57  (-0.94, -0.19) | <.001 |  | 0.10  (-0.29, 0.50) | .61 |  | -1.10  (-1.87, -0.34) | <.001 |
| Sensory | -0.36  (-0.70, 0.02) | .04 |  | 0.15  (-1.17, 1.46) | .83 |  | 0.17  (-0.17, 0.51) | .33 |  | -0.09  (-0.45, 0.26) | .61 |  | -0.44  (-0.82, 0.07) | .02 |  | -0.63  (-1.37, 0.11) | .10 |
| Vitality | 0.12  (-0.14, 0.37) | .37 |  | -0.32  (-1.28, 0.65) | .52 |  | 0  (-0.27, 0.27) | .99 |  | -0.32  (-0.58, -0.06) | .01 |  | 0.13  (-0.14, 0.40) | .33 |  | -0.46  (-0.97, 0.05) | .08 |
| Psychological | -1.74  (-2.15, -1.34) | <.001 |  | -0.63  (-2.08, 0.83) | .40 |  | 0.50  (0.10, 0.91) | .02 |  | 0.56  (0.14, 0.99) | .01 |  | -1.52  (-1.97, -1.07) | <.001 |  | 0.50  (-0.34, 1.34) | .25 |
| Cognitive | -0.28  (-0.54, -0.02) | 0.03 |  | -1.60  (-2.58, -0.61) | <.001 |  | 0.42  (0.17, 0.68) | <.001 |  | 0.41  (0.13, 0.69) | <.001 |  | -0.39  (-0.68, -0.10) | 0.001 |  | -1.05  (-1.62, -0.49) | <.001 |

Multivariate linear regression analysis adjusted 29 covariates including demographic variables (age, gender, residence, education level, and married status )+ socioeconomic status (annual household income) +lifestyle (drinking history, smoking history, physical activity level, memory, mental health, sleep quality ,and tranquilizers or sleeping drugs) +health status (BMI, physical disabilities, hypertension, dyslipidemia, diabetes, cancer, chronic lung diseases, liver disease, heart diseases, stroke, kidney diseases, digestive diseases, psychiatric problems, memory-related diseases, arthritis or rheumatism, and asthma). The IC total score was adjusted using the residual method.

Table S8. The Mediation analysis of 14 chronic diseases and BMI changes between IC and total sleep duration less than 6 hours.

| All-cause | IC total score | |  | Locomotion | |  | Sensory | |  | Vitality | |  | Psychological | |  | Cognitive | |
| --- | --- | --- | --- | --- | --- | --- | --- | --- | --- | --- | --- | --- | --- | --- | --- | --- | --- |
|  | Mediation proportion (%) | *P* Value |  | Mediation proportion (%) | *P* Value |  | Mediation proportion (%) | *P* Value |  | Mediation proportion (%) | *P* Value |  | Mediation proportion (%) | *P* Value |  | Mediation proportion (%) | *P* Value |
|  |  |  |  |  |  |  |  |  |  |  |  |  |  |  |  |  |  |
|  |  |  |  |  |  |  |  |  |  |  |  |  |  |  |  |  |  |
| Hypertension | -0.16 | .73 |  | -0.02 | .95 |  | -0.16 | .78 |  | 0.02 | .96 |  | -0.09 | .71 |  | -0.10 | .78 |
| Dyslipidemia | 0.41 | .453 |  | 0.27 | .87 |  | 1.26 | .50 |  | -0.08 | .91 |  | 0.28 | .53 |  | 0.09 | .67 |
| Diabetes | 1.38 | .09 |  | 0.13 | .96 |  | 1.56 | .37 |  | 1.42 | .76 |  | 1.09 | .04 |  | 0.86 | .28 |
| Cancer | -0.01 | .90 |  | -0.10 | .90 |  | 0.37 | .61 |  | -0.17 | .89 |  | 0.14 | .57 |  | -0.04 | .80 |
| Chronic lung diseases | 0.01 | .94 |  | 0.09 | .93 |  | 0.07 | .84 |  | -0.03 | .97 |  | 0.05 | .75 |  | -0.01 | .93 |
| Liver diseases | -0.01 | .95 |  | -0.04 | .95 |  | 0.86 | .35 |  | -0.09 | .89 |  | 0.01 | .91 |  | 0.01 | .93 |
| Heart diseases | -0.01 | .91 |  | <0.01 | .99 |  | -0.02 | .93 |  | -0.01 | .97 |  | -0.01 | .92 |  | <0.01 | >.99 |
| Stroke | -0.13 | .84 |  | -0.05 | .96 |  | -0.04 | .92 |  | <0.01 | >.99 |  | -0.03 | .89 |  | -0.02 | .92 |
| Kidney diseases | -0.11 | .73 |  | -0.22 | .86 |  | 0.77 | .39 |  | 1.29 | .72 |  | 0.21 | .43 |  | -0.99 | .11 |
| Digestive diseases | 1.21 | .06 |  | -0.53 | .81 |  | 2.81 | .09 |  | 1.24 | .74 |  | 0.46 | .20 |  | 0.71 | .28 |
| Psychiatric problems | 0.16 | .60 |  | 0.05 | .94 |  | -0.10 | .77 |  | 0.05 | .92 |  | 0.37 | .48 |  | 0.15 | .62 |
| Memory-related diseases | 0.39 | .48 |  | 0.07 | .94 |  | 0.68 | .47 |  | 0.45 | .83 |  | 0.25 | .53 |  | 0.23 | .51 |
| Arthritis or rheumatism | 0.37 | .76 |  | -0.01 | .99 |  | 0.39 | .79 |  | 0.13 | .91 |  | 0.27 | .75 |  | 0.25 | .76 |
| Asthma | 0.09 | .91 |  | <0.01 | .99 |  | 0.02 | .93 |  | 0.53 | .91 |  | 0.03 | .85 |  | 0.03 | .90 |
| BMI changes | -0.30 | .41 |  | 0.17 | .86 |  | -0.61 | .44 |  | -1.02 | .76 |  | -0.28 | .30 |  | -0.05 | .78 |

The total effect was determined by the structural equation model (SEMs). The mediation proportion was calculated based on the association between sleep duration and IC (linear model). All models were conducted by adjusting for demographic variables (age, gender, residence, education level, and married status )+ socioeconomic status (annual household income) +lifestyle (drinking history, smoking history, physical activity level, memory, mental health, sleep quality ,and tranquilizers or sleeping drugs) +health status (BMI, physical disabilities, hypertension, dyslipidemia, diabetes, cancer, chronic lung diseases, liver disease, heart diseases, stroke, kidney diseases, digestive diseases, psychiatric problems, memory-related diseases, arthritis or rheumatism, and asthma). The IC total score was adjusted using the residual method.

Table S9. The Mediation analysis of 14 chronic diseases and BMI changes between IC and total sleep duration more than 10 hours.

| All-cause | IC total score | |  | Locomotion | |  | Sensory | |  | Vitality | |  | Psychological | |  | Cognitive | |
| --- | --- | --- | --- | --- | --- | --- | --- | --- | --- | --- | --- | --- | --- | --- | --- | --- | --- |
|  | Mediation proportion (%) | *P* Value |  | Mediation proportion (%) | *P* Value |  | Mediation proportion (%) | *P* Value |  | Mediation proportion (%) | *P* Value |  | Mediation proportion (%) | *P* Value |  | Mediation proportion (%) | *P* Value |
|  |  |  |  |  |  |  |  |  |  |  |  |  |  |  |  |  |  |
|  |  |  |  |  |  |  |  |  |  |  |  |  |  |  |  |  |  |
| Hypertension | -0.05 | .77 |  | 0.01 | .91 |  | -0.11 | .82 |  | 0.13 | .76 |  | 0.07 | .90 |  | -0.12 | .73 |
| Dyslipidemia | -0.39 | .29 |  | -0.56 | .24 |  | -2.52 | .14 |  | 0.83 | .26 |  | 1.08 | .71 |  | -0.12 | .74 |
| Diabetes | -0.42 | .44 |  | -0.35 | .53 |  | -0.34 | .60 |  | -0.17 | .65 |  | 0.75 | .77 |  | -0.24 | .53 |
| Cancer | -0.05 | .85 |  | 0.17 | .42 |  | -1.15 | .25 |  | 0.54 | .39 |  | 1.88 | .59 |  | 0.02 | .93 |
| Chronic lung diseases | -0.08 | .75 |  | -0.22 | .66 |  | -0.13 | .80 |  | 0.31 | .78 |  | 0.31 | .85 |  | -0.10 | .70 |
| Liver diseases | 0.17 | .48 |  | 0.37 | .13 |  | -1.10 | .26 |  | 1.29 | .09 |  | 0.73 | .70 |  | 0.06 | .78 |
| Heart diseases | <0.01 | .97 |  | <0.01 | .97 |  | -0.01 | .97 |  | <0.01 | .97 |  | <0.01 | >.99 |  | <0.01 | .99 |
| Stroke | -0.05 | .79 |  | -0.04 | .82 |  | -0.13 | .80 |  | -0.03 | .90 |  | 0.13 | .89 |  | <0.01 | .99 |
| Kidney diseases | -0.03 | .84 |  | 0.01 | .92 |  | 0.12 | .82 |  | 0.44 | .67 |  | -0.01 | .98 |  | -0.47 | .58 |
| Digestive diseases | -0.26 | .42 |  | 0.04 | .85 |  | -1.09 | .40 |  | -0.64 | .43 |  | 0.95 | .72 |  | <0.01 | >.99 |
| Psychiatric problems | 0.31 | .57 |  | 0.33 | .62 |  | -0.41 | .69 |  | 0.01 | .96 |  | -0.89 | .85 |  | 0.39 | .57 |
| Memory-related diseases | 1.15 | .49 |  | 0.87 | .49 |  | 1.70 | .50 |  | 1.05 | .50 |  | -2.30 | .77 |  | 0.68 | .49 |
| Arthritis or rheumatism | -1.04 | .15 |  | -0.02 | .95 |  | -2.61 | .23 |  | -1.80 | .20 |  | 3.36 | .65 |  | -0.26 | .53 |
| Asthma | -0.43 | .36 |  | -0.04 | .83 |  | -0.16 | .75 |  | -0.94 | .39 |  | -0.02 | .97 |  | -0.17 | .54 |
| BMI changes | -0.04 | .90 |  | 0.04 | .85 |  | -0.07 | .88 |  | -0.13 | .86 |  | 0.12 | .93 |  | -0.05 | .89 |

The total effect was determined by the structural equation model (SEMs). The mediation proportion was calculated based on the association between sleep duration and IC (linear model). All models were conducted by adjusting for demographic variables (age, gender, residence, education level, and married status )+ socioeconomic status (annual household income) +lifestyle (drinking history, smoking history, physical activity level, memory, mental health, sleep quality ,and tranquilizers or sleeping drugs) +health status (BMI, physical disabilities, hypertension, dyslipidemia, diabetes, cancer, chronic lung diseases, liver disease, heart diseases, stroke, kidney diseases, digestive diseases, psychiatric problems, memory-related diseases, arthritis or rheumatism, and asthma). The IC total score was adjusted using the residual method.

Table S10. The Mediation analysis of 14 chronic diseases and BMI changes between IC and moderate nap duration less than 60 minutes.

| All-cause | IC total score | |  | Locomotion | |  | Sensory | |  | Vitality | |  | Psychological | |  | Cognitive | |
| --- | --- | --- | --- | --- | --- | --- | --- | --- | --- | --- | --- | --- | --- | --- | --- | --- | --- |
|  | Mediation proportion (%) | *P* Value |  | Mediation proportion (%) | *P* Value |  | Mediation proportion (%) | *P* Value |  | Mediation proportion (%) | *P* Value |  | Mediation proportion (%) | *P* Value |  | Mediation proportion (%) | *P* Value |
|  |  |  |  |  |  |  |  |  |  |  |  |  |  |  |  |  |  |
|  |  |  |  |  |  |  |  |  |  |  |  |  |  |  |  |  |  |
| Hypertension | -1.64 | .10 |  | -0.34 | .52 |  | -1.49 | .54 |  | -0.32 | .67 |  | -1.17 | .16 |  | -1.39 | .12 |
| Dyslipidemia | -3.37 | .02 |  | 3.13 | .05 |  | -8.94 | .45 |  | -0.25 | .90 |  | -4.95 | <.01 |  | 0.25 | .80 |
| Diabetes | -0.25 | .76 |  | 0.12 | .78 |  | -0.05 | .92 |  | -0.18 | .82 |  | -0.24 | .77 |  | -0.05 | .82 |
| Cancer | 0.09 | .73 |  | -0.30 | .36 |  | -0.90 | .62 |  | 0.89 | .45 |  | -0.58 | .37 |  | 0.20 | .52 |
| Chronic lung diseases | 0.03 | .86 |  | -0.14 | .69 |  | 0.22 | .80 |  | -0.30 | .79 |  | 0.23 | .56 |  | 0.02 | .90 |
| Liver diseases | -0.01 | .91 |  | 0.02 | .89 |  | -0.21 | .89 |  | <0.01 | .98 |  | -0.03 | .87 |  | <0.01 | .97 |
| Heart diseases | -0.34 | .56 |  | 0.59 | .34 |  | -0.39 | .75 |  | -0.41 | .63 |  | -0.43 | .45 |  | <0.01 | .98 |
| Stroke | -0.91 | .24 |  | 1.24 | .26 |  | 0.16 | .87 |  | -0.64 | .58 |  | -0.70 | .27 |  | -0.17 | .64 |
| Kidney diseases | -0.02 | .91 |  | -0.20 | .52 |  | -0.48 | .68 |  | -0.68 | .53 |  | -0.46 | .46 |  | 0.14 | .63 |
| Digestive diseases | -1.48 | .17 |  | -0.79 | .24 |  | -2.59 | .51 |  | -0.71 | .52 |  | -0.36 | .47 |  | -1.29 | .17 |
| Psychiatric problems | 0.04 | .85 |  | -0.19 | .65 |  | -0.07 | .89 |  | -0.03 | .90 |  | 0.53 | .52 |  | 0.01 | .93 |
| Memory-related diseases | 0.16 | .83 |  | <0.01 | .99 |  | 0.17 | .89 |  | 0.03 | .89 |  | 0.23 | .70 |  | 0.18 | .78 |
| Arthritis or rheumatism | 0.49 | .78 |  | -0.05 | .84 |  | 0.47 | .84 |  | 0.10 | .86 |  | 0.54 | .76 |  | 0.28 | .77 |
| Asthma | -1.84 | .07 |  | -0.11 | .83 |  | -0.53 | .73 |  | -4.55 | .23 |  | -0.82 | .27 |  | -0.48 | .37 |
| BMI changes | 0.03 | .89 |  | 0.04 | .83 |  | 0.44 | .79 |  | -0.43 | .83 |  | 0.19 | .65 |  | 0.05 | .78 |

The total effect was determined by the structural equation model (SEMs). The mediation proportion was calculated based on the association between sleep duration and IC (linear model). All models were conducted by adjusting for demographic variables (age, gender, residence, education level, and married status )+ socioeconomic status (annual household income) +lifestyle (drinking history, smoking history, physical activity level, memory, mental health, sleep quality ,and tranquilizers or sleeping drugs) +health status (BMI, physical disabilities, hypertension, dyslipidemia, diabetes, cancer, chronic lung diseases, liver disease, heart diseases, stroke, kidney diseases, digestive diseases, psychiatric problems, memory-related diseases, arthritis or rheumatism, and asthma). The IC total score was adjusted using the residual method.

Table S11. The Mediation analysis of 14 chronic diseases and BMI changes between IC and nap duration more than 60 minutes.

| All-cause | IC total score | |  | Locomotion | |  | Sensory | |  | Vitality | |  | Psychological | |  | Cognitive | |
| --- | --- | --- | --- | --- | --- | --- | --- | --- | --- | --- | --- | --- | --- | --- | --- | --- | --- |
|  | Mediation proportion (%) | *P* Value |  | Mediation proportion (%) | *P* Value |  | Mediation proportion (%) | *P* Value |  | Mediation proportion (%) | *P* Value |  | Mediation proportion (%) | *P* Value |  | Mediation proportion (%) | *P* Value |
|  |  |  |  |  |  |  |  |  |  |  |  |  |  |  |  |  |  |
|  |  |  |  |  |  |  |  |  |  |  |  |  |  |  |  |  |  |
| Hypertension | -0.67 | .53 |  | 0.03 | .83 |  | -0.11 | .95 |  | <0.01 | .99 |  | -0.69 | .35 |  | -0.07 | .76 |
| Dyslipidemia | -2.34 | .24 |  | 0.13 | .74 |  | -1.23 | .92 |  | -0.57 | .64 |  | -1.75 | .06 |  | -0.64 | .30 |
| Diabetes | -0.97 | .53 |  | 0.09 | .69 |  | -0.08 | .95 |  | -0.20 | .75 |  | -0.96 | .41 |  | -0.31 | .49 |
| Cancer | <0.01 | >.99 |  | <0.01 | .97 |  | 0.02 | .99 |  | 0.11 | .89 |  | 0.05 | .87 |  | <0.01 | .96 |
| Chronic lung diseases | -1.55 | .35 |  | 0.65 | .23 |  | -0.39 | .94 |  | 1.07 | .45 |  | -0.80 | .26 |  | -0.06 | .82 |
| Liver diseases | -0.02 | .94 |  | <0.01 | .97 |  | 0.01 | .98 |  | 0.03 | .95 |  | -0.01 | .95 |  | <0.01 | .98 |
| Heart diseases | -0.25 | .78 |  | 0.02 | .93 |  | 0.01 | .99 |  | -0.34 | .72 |  | -0.41 | .48 |  | -0.09 | .80 |
| Stroke | -2.14 | .33 |  | 0.60 | .24 |  | 0.05 | .97 |  | 2.02 | .37 |  | -0.77 | .27 |  | -0.48 | .33 |
| Kidney diseases | -0.01 | .97 |  | <0.01 | .95 |  | 0.02 | .98 |  | 0.04 | .90 |  | -0.06 | .85 |  | 0.04 | .88 |
| Digestive diseases | 0.14 | .90 |  | <0.01 | .99 |  | 0.09 | .98 |  | <0.01 | .98 |  | 0.07 | .90 |  | 0.02 | .92 |
| Psychiatric problems | -1.25 | .51 |  | 0.17 | .56 |  | <0.01 | >.99 |  | 0.19 | .75 |  | -0.90 | .40 |  | -0.74 | .36 |
| Memory-related diseases | -2.03 | .38 |  | 0.43 | .36 |  | -0.06 | .97 |  | 0.68 | .57 |  | -0.95 | .26 |  | -0.63 | .28 |
| Arthritis or rheumatism | 4.85 | .19 |  | 0.23 | .53 |  | 1.11 | .93 |  | -4.05 | .28 |  | 3.64 | .05 |  | >0.99 | .15 |
| Asthma | -1.87 | .31 |  | -0.18 | .59 |  | -0.04 | .97 |  | 4.61 | .35 |  | -0.92 | .23 |  | -0.01 | .95 |
| BMI changes | 1.52 | .54 |  | 0.01 | .93 |  | 0.14 | .94 |  | -1.07 | .62 |  | 0.64 | .48 |  | 0.40 | .49 |

The total effect was determined by the structural equation model (SEMs). The mediation proportion was calculated based on the association between sleep duration and IC (linear model). All models were conducted by adjusting for demographic variables (age, gender, residence, education level, and married status )+ socioeconomic status (annual household income) +lifestyle (drinking history, smoking history, physical activity level, memory, mental health, sleep quality ,and tranquilizers or sleeping drugs) +health status (BMI, physical disabilities, hypertension, dyslipidemia, diabetes, cancer, chronic lung diseases, liver disease, heart diseases, stroke, kidney diseases, digestive diseases, psychiatric problems, memory-related diseases, arthritis or rheumatism, and asthma). The IC total score was adjusted using the residual method.

Table S12. The Mediation analysis of 14 chronic diseases and BMI changes between IC and nighttime sleep duration less than 6 hours.

| All-cause | IC total score | |  | Locomotion | |  | Sensory | |  | Vitality | |  | Psychological | |  | Cognitive | |
| --- | --- | --- | --- | --- | --- | --- | --- | --- | --- | --- | --- | --- | --- | --- | --- | --- | --- |
|  | Mediation proportion (%) | *P* Value |  | Mediation proportion (%) | *P* Value |  | Mediation proportion (%) | *P* Value |  | Mediation proportion (%) | *P* Value |  | Mediation proportion (%) | *P* Value |  | Mediation proportion (%) | *P* Value |
|  |  |  |  |  |  |  |  |  |  |  |  |  |  |  |  |  |  |
|  |  |  |  |  |  |  |  |  |  |  |  |  |  |  |  |  |  |
| Hypertension | -0.34 | .42 |  | -0.29 | .63 |  | -0.58 | .45 |  | -0.03 | .95 |  | -0.17 | .40 |  | -0.27 | .55 |
| Dyslipidemia | 0.63 | .26 |  | >0.99 | .42 |  | 1.85 | .30 |  | -0.13 | .87 |  | 0.36 | .27 |  | 0.23 | .58 |
| Diabetes | 0.76 | .14 |  | 0.32 | .78 |  | 1.24 | .28 |  | -0.02 | .99 |  | 0.63 | .05 |  | 0.87 | .25 |
| Cancer | -0.01 | .91 |  | 0.13 | .71 |  | -0.30 | .68 |  | 0.07 | .93 |  | -0.12 | .61 |  | 0.01 | .93 |
| Chronic lung diseases | 0.05 | .75 |  | 0.48 | .64 |  | 0.11 | .74 |  | -0.06 | .94 |  | 0.11 | .57 |  | -0.02 | .89 |
| Liver diseases | <0.01 | .96 |  | <0.01 | .97 |  | -0.13 | .85 |  | 0.01 | .98 |  | 0.00 | .94 |  | 0.00 | .98 |
| Heart diseases | 0.01 | .89 |  | 0.03 | .89 |  | <0.01 | .99 |  | 0.01 | .97 |  | 0.02 | .82 |  | 0.01 | .94 |
| Stroke | 0.27 | .65 |  | 0.52 | .69 |  | 0.15 | .72 |  | 0.22 | .89 |  | 0.09 | .63 |  | 0.10 | .73 |
| Kidney diseases | -0.12 | .81 |  | -0.50 | .62 |  | 1.24 | .25 |  | 0.94 | .77 |  | 0.40 | .17 |  | -1.70 | .09 |
| Digestive diseases | 1.89 | .02 |  | -1.19 | .58 |  | 3.89 | .06 |  | 1.88 | .78 |  | 0.64 | .13 |  | 1.93 | .13 |
| Psychiatric problems | 0.57 | .23 |  | 0.72 | .53 |  | -0.37 | .60 |  | 0.34 | .84 |  | 0.55 | .10 |  | 0.91 | .22 |
| Memory-related diseases | 0.03 | .91 |  | -0.01 | .97 |  | 0.03 | .93 |  | 0.01 | .97 |  | 0.02 | .96 |  | 0.03 | .89 |
| Arthritis or rheumatism | -0.41 | .72 |  | -0.02 | .91 |  | -0.32 | .77 |  | -0.10 | .95 |  | -0.21 | .75 |  | -0.38 | .75 |
| Asthma | -0.74 | .28 |  | <0.01 | .99 |  | -0.21 | .64 |  | -2.04 | .80 |  | -0.17 | .40 |  | -0.27 | .53 |
| BMI changes | -0.09 | .83 |  | 0.02 | .90 |  | -0.14 | .81 |  | -0.07 | .97 |  | -0.05 | .82 |  | -0.02 | .88 |

The total effect was determined by the structural equation model (SEMs). The mediation proportion was calculated based on the association between sleep duration and IC (linear model). All models were conducted by adjusting for demographic variables (age, gender, residence, education level, and married status )+ socioeconomic status (annual household income) +lifestyle (drinking history, smoking history, physical activity level, memory, mental health, sleep quality ,and tranquilizers or sleeping drugs) +health status (BMI, physical disabilities, hypertension, dyslipidemia, diabetes, cancer, chronic lung diseases, liver disease, heart diseases, stroke, kidney diseases, digestive diseases, psychiatric problems, memory-related diseases, arthritis or rheumatism, and asthma). The IC total score was adjusted using the residual method.

Table S13. The Mediation analysis of 14 chronic diseases and BMI changes between IC and nighttime sleep duration more than 10 hours.

| All-cause | IC total score | |  | Locomotion | |  | Sensory | |  | Vitality | |  | Psychological | |  | Cognitive | |
| --- | --- | --- | --- | --- | --- | --- | --- | --- | --- | --- | --- | --- | --- | --- | --- | --- | --- |
|  | Mediation proportion (%) | *P* Value |  | Mediation proportion (%) | *P* Value |  | Mediation proportion (%) | *P* Value |  | Mediation proportion (%) | *P* Value |  | Mediation proportion (%) | *P* Value |  | Mediation proportion (%) | *P* Value |
|  |  |  |  |  |  |  |  |  |  |  |  |  |  |  |  |  |  |
|  |  |  |  |  |  |  |  |  |  |  |  |  |  |  |  |  |  |
| Hypertension | -0.04 | .88 |  | -0.02 | .90 |  | 0.19 | .89 |  | 0.02 | .95 |  | -0.03 | .94 |  | <0.01 | .98 |
| Dyslipidemia | -0.28 | .62 |  | -0.53 | .24 |  | 2.60 | .60 |  | 1.14 | .57 |  | -0.46 | .73 |  | 0.32 | .55 |
| Diabetes | 0.12 | .84 |  | 0.26 | .76 |  | -0.04 | .95 |  | -0.06 | .93 |  | 0.50 | .834 |  | 0.06 | .85 |
| Cancer | -0.20 | .40 |  | 0.06 | .63 |  | 0.56 | .61 |  | 0.23 | .75 |  | -1.24 | .36 |  | -0.08 | .70 |
| Chronic lung diseases | 0.08 | .89 |  | 0.21 | .84 |  | -0.10 | .95 |  | -0.03 | .97 |  | 0.48 | .84 |  | 0.02 | .93 |
| Liver diseases | 0.03 | .93 |  | 0.19 | .45 |  | 0.96 | .65 |  | 1.03 | .55 |  | -1.31 | .40 |  | 0.09 | .81 |
| Heart diseases | -0.49 | .54 |  | -0.16 | .75 |  | 0.13 | .95 |  | -0.08 | .97 |  | -0.30 | .87 |  | -0.30 | .67 |
| Stroke | 0.04 | .97 |  | -0.01 | .98 |  | 0.01 | .97 |  | 0.01 | .99 |  | 0.05 | .96 |  | <0.01 | .98 |
| Kidney diseases | 0.04 | .89 |  | 0.01 | .89 |  | 0.01 | .96 |  | 0.01 | .97 |  | <0.01 | >.99 |  | 0.12 | .86 |
| Digestive diseases | -0.17 | .73 |  | 0.01 | .96 |  | 0.52 | .82 |  | -0.17 | .86 |  | -0.25 | .82 |  | <0.01 | .99 |
| Psychiatric problems | 1.17 | .56 |  | 0.91 | .59 |  | 0.46 | .84 |  | 1.13 | .74 |  | 1.25 | .73 |  | 1.04 | .52 |
| Memory-related diseases | 0.46 | .72 |  | 0.04 | .89 |  | -0.07 | .92 |  | 0.09 | .90 |  | 1.04 | .80 |  | 0.35 | .72 |
| Arthritis or rheumatism | -1.30 | .40 |  | -0.10 | .70 |  | 1.02 | .74 |  | -1.15 | .73 |  | -2.27 | .65 |  | -0.34 | .55 |
| Asthma | -2.28 | .04 |  | -0.91 | .27 |  | 1.12 | .69 |  | -5.98 | .53 |  | -0.32 | .86 |  | -0.48 | .52 |
| BMI changes | 0.33 | .72 |  | -0.02 | .84 |  | -0.23 | .87 |  | 0.31 | .85 |  | 0.22 | .82 |  | 0.20 | .73 |

The total effect was determined by the structural equation model (SEMs). The mediation proportion was calculated based on the association between sleep duration and IC (linear model). All models were conducted by adjusting for demographic variables (age, gender, residence, education level, and married status )+ socioeconomic status (annual household income) +lifestyle (drinking history, smoking history, physical activity level, memory, mental health, sleep quality, and tranquilizers or sleeping drugs) +health status (BMI, physical disabilities, hypertension, dyslipidemia, diabetes, cancer, chronic lung diseases, liver disease, heart diseases, stroke, kidney diseases, digestive diseases, psychiatric problems, memory-related diseases, arthritis or rheumatism, and asthma). The IC total score was adjusted using the residual method.
